# Atomistic-Level Insights into the Role of Mutations in the Engineering of PET Hydrolases: A Systematic Review

**DOI:** 10.3390/ijms26167682

**Published:** 2025-08-08

**Authors:** Athina Karaoli, Haralampos Tzoupis, Konstantinos D. Papavasileiou, Anastasios G. Papadiamantis, Dimitris G. Mintis, Chris T. Kiranoudis, Iseult Lynch, Georgia Melagraki, Antreas Afantitis

**Affiliations:** 1 Department of ChemInformatics, NovaMechanics Ltd., Nicosia 1070, Cyprus; karaoli@novamechanics.com (A.K.); tzoupis@novamechanics.com (H.T.); papavasileiou@novamechanics.com (K.D.P.); papadiamantis@novamechanics.com (A.G.P.); mintis@novamechanics.com (D.G.M.); 2 School of Chemical Engineering, National Technical University, Zografou, 15780 Athens, Greece; kyr@chemeng.ntua.gr; 3 Department of ChemInformatics, NovaMechanics MIKE, 18545 Piraeus, Greece; 4 Entelos Institute, Nicosia 2102, Cyprus; i.lynch@bham.ac.uk; 5 School of Geography, Earth and Environmental Sciences, University of Birmingham, Birmingham B15 2TT, UK; 6 Division of Physical Sciences and Applications, Hellenic Military Academy, 16672 Vari, Greece; georgiamelagraki@gmail.com

**Keywords:** plastics, poly(ethylene) terephthalate, PET, enzymatic degradation, molecular dynamics, site-directed mutagenesis

## Abstract

Plastic pollution is a growing global challenge, and traditional plastic waste management methods are proving inadequate in tackling the issue. Enzymatic biodegradation has emerged as a promising alternative or addition to plastic waste management due to its environmentally friendly profile. Polyethylene terephthalate (PET) is among the most widely used polymers in packaging, and recent research has identified several PET-degrading enzymes, such as TfCut2, *Is*PETase, and LCC, as promising candidates for biodegradation applications at the industrial level. This has led to extensive efforts to improve their catalytic efficiency, with targeted mutagenesis being the preferred method employed for their modification. To this end, molecular dynamics (MD) simulations coupled with experimental validation have provided critical atomistic-level insights into the effect of mutations on enzymatic function. The present systematic review examines the role of mutations in determining enzymatic activity and thermostability, analyzing their structural and mechanistic contributions across 20 studies. The integration of MD simulations and experimental findings allows elucidation of the mechanistic details governing polymer degradation, as well as identification of key residue and enzyme hotspots that enhance catalytic performance. The review further highlights the role of MD simulations as powerful tools in providing valuable insights to guide targeted mutations for enzyme efficiency optimization.

## 1. Introduction

The large-scale production of synthetic polymers was initiated in the 1950s [1] and has since become one of the most significant environmental challenges currently affecting modern societies. In 2023 alone, the global production of plastics reached 413.8 million tons, with the vast majority (91.3%) consisting of polymers such as polyethylene (PE), polyethylene terephthalate (PET), polyurethane (PUR), polystyrene (PS), polypropylene (PP), and polyvinyl chloride (PVC), and only 8.7% were derived from recycled sources [2]. The main characteristic of these materials is their high resistance to degradation under natural conditions, which contributes substantially to plastic waste and environmental pollution [3]. However, despite their resistance, long-term exposure to environmental weathering can lead to their slow physical fragmentation into micro- and nanoplastics [4,5]. These degradation products accumulate in landfills [6], soils [6], marine environments [7], and in groundwater [8]. Moreover, these micro- and nanoplastics have been thoroughly documented to have entered the food chain [9], having also been detected in seafood [10] as well as drinking water [11]. The consumption of food and water contaminated with plastics may potentially pose significant risks to human health, although the full extent remains unknown [9].

Traditional methods for managing plastic waste, such as incineration (24% of plastic waste) and landfilling (58% of plastic waste), have proven insufficient to address the growing scale of plastic contamination [12]. The incineration of plastics leads to secondary environmental problems, as it releases toxic substances [13] such as heavy metals, polychlorinated dibenzodioxins (PCDD), polychlorinated dibenzofurans (PCDF), polycyclic aromatics (PCA) [14], which pollutes groundwater and poses significant health risks [13], and greenhouse gases such CO_2_ [15]. Plastics, when recycled, are either converted into other products through mechanical recycling or undergo chemical processing and are broken down into their constituent monomers or oligomers that can then be used to produce other synthetic chemicals [16]. While mechanical recycling reduces the quality of recycled plastics [17], chemical recycling via thermal and catalytic degradation (e.g., pyrolysis, which requires high temperatures of >300 °C) is very energy-intensive [15,18].

In this context, enzymatic biodegradation offers a promising and eco-friendly alternative for tackling plastic pollution. Microbes, such as bacteria, fungi, and actinomycetes [19,20], are able to employ enzymes that break down the polymeric structure of plastics. This process could potentially convert plastic waste either into reusable materials or biomass, offering a sustainable alternative to plastic waste management [21]. Cutinases, lipases, and esterases are among the most common enzymes that have been associated with plastic degradation [19]. The first PET-hydrolyzing enzyme *Thermobifida fusca* hydrolase (TfH) was isolated in 2005 from *Thermobifida fusca* DSM43793 and was found to be capable of breaking down PET polymers [22]. Since then, many PET-degrading enzymes have been isolated from different microorganisms. However, their applicability is limited at ambient conditions as their optimal activity typically requires higher temperatures, around 70 °C [23], near the glass transition temperature of PET [24]. A major breakthrough was made in 2016 with the isolation of the bacterium *Ideonella sakaiensis* 201-F6, which is capable of degrading PET using two enzymes: *Is*PETase and MHETase [25]. These two enzymes significantly contributed to accelerating the PET degradation rate, even at moderate temperatures. However, *Is*PETase is sensitive to higher temperatures, and as a result its activity is relatively short-lived, lasting around 24 h at 37 °C [26]. Since then, various studies have focused on understanding the enzymatic pathways and catalytic mechanisms [27,28] involved in PET degradation, with the aim of improving enzyme efficiency, durability, and thermostability [23,29,30,31,32].

Although experimental approaches are crucial for verifying plastic degradation, computational methods have gained significant attention for studying degradation processes in recent years [33]. Molecular dynamics (MD) simulations along with protein-ligand docking techniques have been employed to analyze the interactions between enzymes and plastic polymers at the atomistic level [34,35]. Molecular docking models are used to predict enzyme binding modes to plastic polymers, highlighting key residues involved in catalysis, while MD simulations enable the analysis of these systems over time, ranging from picoseconds to microseconds, providing insights into how the enzyme’s conformation changes during the catalytic process [36,37]. These simulations facilitate the study of residue interactions that affect ligand binding, offering a deeper understanding of the molecular mechanisms underlying plastic polymer breakdown. Additionally, MD facilitates the rational design and engineering of enzymes with enhanced thermostability and catalytic activity [38,39] by identifying specific regions within the enzyme that are catalytically important. The results can be implemented for site-directed mutagenesis experiments, aiding in the strategic optimization of enzymatic function through targeted modifications [40].

In recent years, numerous literature reviews have focused on the enzymatic degradation of PET [41,42,43,44,45] with emphasis on *Is*PETase [46], primarily assessing laboratory experiments. Notably, the reviews by Liu et al. (2023) and Barclay et al. (2023) provide a comprehensive analysis of *Is*PETase’s structural characteristics, ligand–protein interactions, and the impact of mutations on its activity and stability [46,47]. More specifically, Liu et al. (2023) [46] discussed the impact of mutations on *Is*PETase activity and stability, emphasizing experimental findings, with MD simulation results included only in a limited, supportive role. Barclay et al. (2023) [47], on the other hand, focused primarily on molecular docking simulations to understand substrate positioning within the active site, offering a largely static structural perspective of *Is*PETase. The scope of the present systematic review is to analyze and assess MD findings that enable a time-resolved understanding of amino acid behavior within the local environment of the enzymes. Importantly, it expands the scope beyond *Is*PETase to include a wide range of PET-degrading enzymes compared to the other two studies. The application of MD simulations in combination with experimental processes can provide valuable information regarding the flexibility of structural loops and binding affinities, leading to deeper insights into enzyme functionality.

## 2. Materials and Methods

### 2.1. Protocol and Registration

This systematic review was registered on the Open Science Framework (OSF) Repository (https://doi.org/10.17605/OSF.IO/8JXAV, accessed on 29 May 2025). The research question was formulated as follows: “What mechanistic insights can MD simulations provide to facilitate the understanding of experimental observations and the impact of mutations on enzyme thermostability and catalytic activity?”. The methodology applied follows the Preferred Reporting Items for Systematic Reviews and Meta-Analyses (PRISMAs) checklist (Table S1 in the Support Information file) [48].

### 2.2. Eligibility Criteria

The research studies found in the initial search were selected for inclusion only if they satisfied the following criteria:

(i) examined the enzymatic breakdown of PET using proteins bioengineered through site-directed mutagenesis;
(ii) combined experimental results and MD simulations;
(iii) were written in English.

Studies were excluded if they matched the following criteria:

(i) involved plastic polymer substrates other than PET;
(ii) focused on microbial degradation, genomics, chemical catalysis, and photocatalysis;
(iii) examined the weathering of plastics and toxicity;
(iv) incorporated only experimental or only computational approaches;
(v) included only other bioengineering strategies (e.g., protein fusion technology, cell surface display, etc.).

### 2.3. Information Sources and Search Strategy

The literature search was conducted using the bibliographic databases PubMed [49] and Scopus [50]. These databases were selected for their extensive peer-reviewed literature as well as their exclusion of the gray literature (e.g., dissertation theses, reports, and consultancy documents) ensuring that only original, peer-reviewed articles were included to maintain a high level of reliability [51]. Moreover, both databases support Boolean operators (AND, OR, and NOT) that allow for efficient search queries, providing high precision and recall [51], thus aiding in appropriately defining the required search terms. Each database has a different interface and indexing methods, such as parentheses and quotation marks; as a result, the search query was adjusted accordingly. Details of the extended search query, post-query filters, and total numbers of results for each database are provided in Table S2. All the articles were written in English. The search on the databases was finalized in September 2024.

The keywords and search terms were the following:

1. “PET” OR “polyethylene terephthalate” OR “poly(ethylene terephthalate)” AND;
2. protein OR enzym* AND;
3. biodegrad* OR degrad* OR depolymeriz* OR bioconversion AND;
4. hydrol* OR cataly* AND;
5. plastic.

The search query was formulated based on research questions focused on the enzymatic degradation of PET. The initial query terms (points 1–3) resulted in a large number of papers (>2000) highlighting the lack of specificity in our query. The scope of adding additional terms (points 4–5) was to increase the sensitivity of our search while avoiding overly specific keywords that could lead to the exclusion of the relevant literature. Moreover, specific keywords indicating the bioengineering method of site-directed mutagenesis and the type of the study were not implemented in the queries to avoid an overly precise query result.

### 2.4. Screening and Selection Process

The titles and abstracts of papers identified using the search terms outlined in Section 2.3 (Table S2) were retrieved from the PubMed and Scopus databases. At the initial stage, items not classified as original research articles (e.g., books and book chapters, conference papers, reviews, short surveys, editorials, erratum, notes, etc.) were excluded. In the following step, all duplicates records were removed with the Rayyan software which is available online (https://www.rayyan.ai/, access date: 7 October 2024) [52]. Then, the keywords, abstracts, and titles of these papers were screened, and the studies meeting the exclusion criteria described in Section 2.2 were excluded. The final stage involved full-text screening, and any studies fulfilling the exclusion criteria were also removed. The management of the articles, including the process of removing duplicate records and abstracts and full-text screening, was optimized with the use of the Rayyan software. The entire selection process was carried out by two reviewers (A.K. and H.T.).

## 3. Results

### 3.1. Literature Search

**Figure 1:**
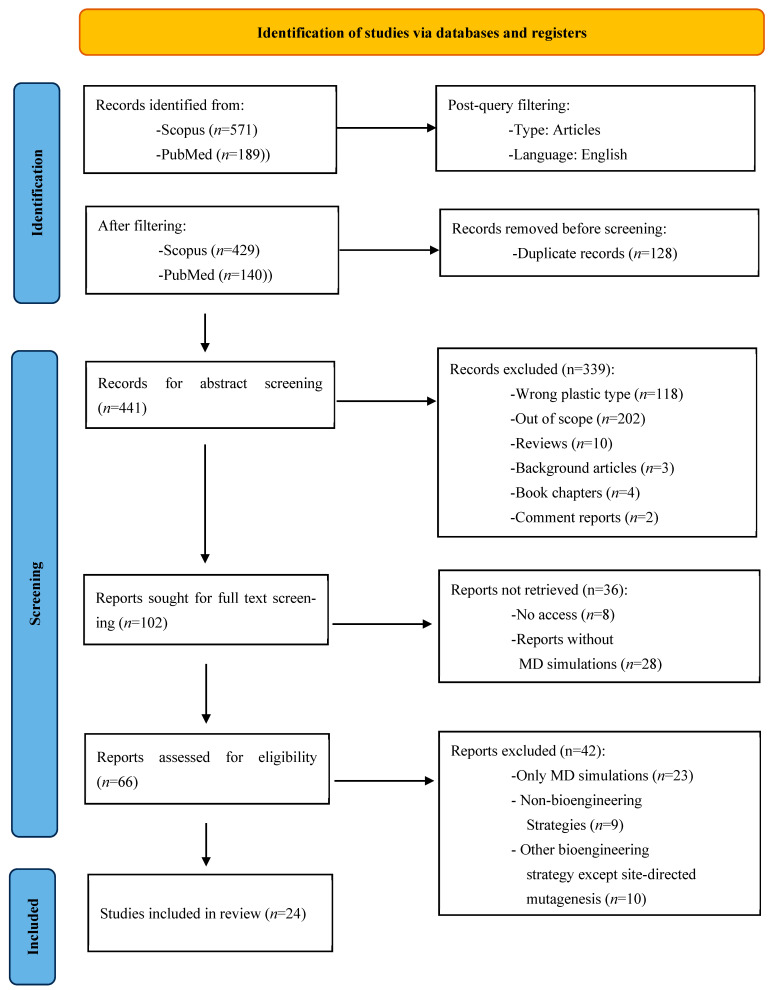
PRISMA 2020 flow diagram of the eligibility assessment process applied in the current systematic review.

This systematic review aims to highlight the interconnection between experimental and computational investigations of enzymatic degradation of plastic, demonstrating how their combination enhances knowledge of plastic biodegradation mechanisms. By combining in vitro and in silico methods, researchers gain a more thorough understanding of the structure–function relationships in engineered enzymes, thereby facilitating rational design and efficient optimization of plastic-degrading enzymes with improved breakdown capabilities and performance. Initially, a total of 760 literature records (571 from Scopus and 189 from PubMed) were identified, as demonstrated in the PRISMA flow diagram (Figure 1). Following an initial screening process, 569 articles were identified as potentially relevant based on their focus on PET biodegradation using engineered enzymes. As illustrated in Figure 1, 128 duplicate entries were removed, resulting in 441 unique articles. These 441 records underwent title and abstract screening, from which 102 studies were selected for full-text evaluation. During this phase, the following occurred:

- 8 articles were excluded due to inaccessible full texts;
- 28 articles employed computational methods that extended beyond the scope of MD simulations (e.g., Monte Carlo approaches, Markov models, quantum mechanics, or static molecular docking);
- 23 articles utilized MD simulations but lacked any accompanying experimental validation;
- 9 studies did not involve any protein engineering strategy;
- 10 studies applied alternative bioengineering techniques (e.g., protein fusion and surface display) not aligned with the site-directed mutagenesis criterion.

After applying the inclusion and exclusion criteria detailed in Section 2, 24 studies were finally identified that integrated MD simulations with experimental validation of site-directed mutagenesis on PET-degrading enzymes, which formed the core dataset analyzed in this review.

### 3.2. Characteristics of the Included Studies

Qualitative analysis of the 24 eligible studies indicates that the earliest published article was in 2015, which indicates the relatively recent adoption of MD simulations in PET enzyme engineering. The majority of the selected articles (62%) were published between January 2023 and July 2024. This highlights the growing implementation of MD simulations to enhance the experimental assessment of plastic biodegradation. Among the 24 articles reviewed, 4 were excluded as the MD simulations, and the experiments performed did not provide further insights into the role of mutations or the improvement in the mechanism of catalysis [53,54,55,56]. The results from the 20 papers included in this study are presented in Table 1. The enzymes under investigation originate from various sources that include thermophilic actinobacteria (e.g., *Thermobifida fusca*), marine bacteria (e.g., *Vibrio gazogenes*), and environmental isolates such as *Ideonella sakaiensis*. Apart from the naturally occurring PETases, bioengineered enzymes, like V3 PETase, were also considered. Wild-type enzymes serve as a template for site-directed mutagenesis, and MD simulations were utilized to examine conformational changes at the atomistic level. Overall, the enzymes presented in Table 1 cover a broad range and allow for comparative insights into the effects of site-directed mutations across various structural backgrounds. In the following sections, the experimental and computational findings are examined in detail, with a focus on structure–function correlations, mutational hotspots, and mechanistic patterns.

### 3.3. Catalytic Mechanism of PET Degradation

**Figure 2:**
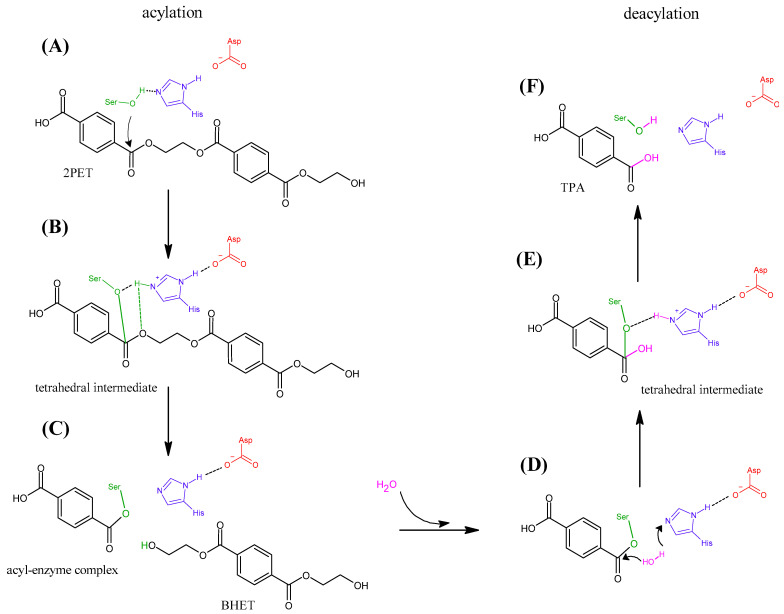
The catalytic degradation of a PET dimer is driven by the catalytic triad—Ser (green), His (purple), and Asp (red)—present in PET-degrading cutinases and positioned in close proximity. The figure depicts both the acylation and diacylation processes, each with its intermediate steps [44,80]. (**A**) Nucleophilic attack on the ester bond; (**B**) Stabilization of the tetrahedral intermediate; (**C**) Formation of an acyl-enzyme complex and release of the first product; (**D**) A water molecule initiates the deacylation phase; (**E**) Formation of a second tetrahedral intermediate; (**F**) Release of the final product.

The reviewed articles focus on the catalytic mechanism of PET degradation via polymer chain breakdown. Specifically, the reaction involves the hydrolysis of the ester bond between PET moieties in the polymer chain, leading to the formation of intermediate products such as mono(2-hydroxyethyl) terephthalate (MHET) and bis(2-hydroxyethyl) terephthalate (BHET). These intermediates can be further degraded into the original PET monomers, terephthalic acid (TPA) and ethylene glycol (EG) [44]. All the enzymes reviewed in this study belong to the family of hydrolases [3], and their catalytic mechanism comprises the amino acids Ser, His, and Asp (Figure 2) [76]. The catalytic reaction is generally distinguished by two processes: acylation and deacylation [27,77,78,79]. During the acylation step, Ser acts as a nucleophile, initiating the attack on the ester bond (Figure 2A) and the formation of a tetrahedral intermediate, while His and Asp stabilize the reaction intermediate and facilitate the proton transfer (Figure 2B) [36]. In the next step, the tetrahedral intermediate breaks, and an acyl-enzyme complex is formed, in which the substrate remains bound to Ser and the first intermediate (BHET) is released (Figure 2C). In the deacylation step, a water molecule initiates the reaction by binding to the acyl-complex (Figure 2D). The hydroxyl group attaches to the substrate forming a tetrahedral intermediate, while a hydrogen is transferred to His (Figure 2E). In the last step, the second tetrahedral intermediate collapses, resulting in the release of the product (TPA) (Figure 2F). Through a similar process, BHET can also be degraded into TPA and EG.

**Figure 3:**
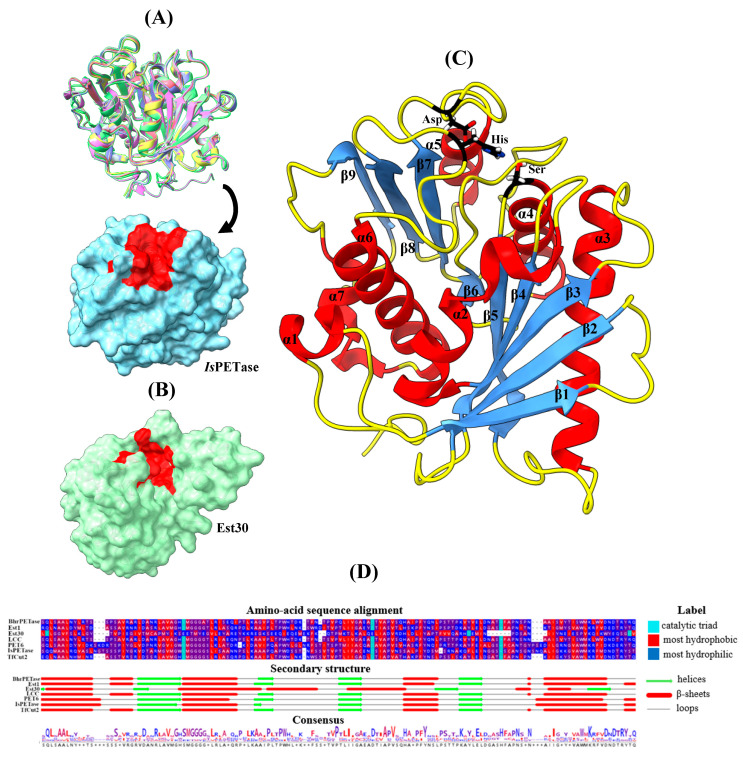
(**A**) Superimposition of the crystal structures of six PET-degrading enzymes: BhrPETase (blue), PET6 (pink), LCC (yellow), *Is*PETase (green), Est1 (grey), and TfCut2 (red). Similar secondary structure elements (cyan) and the active site (red) are also illustrated. (**B**) Crystal structure of Est30. (**C**) Tertiary structure of *Is*PETase with the catalytic triad (black). (**D**) Amino-acid sequence alignment of the seven enzymes using the Clustal algorithm [86]. The residues of the catalytic triad are colored cyan. All residues are colored based on their hydrophobicity index, with the most hydrophobic residues in red and the most hydrophilic ones in blue. Secondary structure elements are illustrated as red barrels for helices and green arrows for β-sheets. The consensus bar graph for the specific region is also provided. The sequence alignment, along with the consensus bars, were produced using Jalview software v2.11.4.0. [87].

The crystallographic structures of the PET-degrading enzymes presented in this review are illustrated in Figure 3, along with their residue alignments and the respective secondary structure elements. Specifically, superposition of six PET-degrading enzymes, extracted from the RCSB Protein Data Bank (PDB), BhrPETase (PDB ID: 7EOA) [81], PET6 (PDB ID: 7Z6B) [63], LCC (PDB ID: 4EBO) [82], *Is*PETase (PDB ID: 6EQE) [25], Est1 (PDB ID: 3WYN) [83], and TfCut2 (PDB ID:4CG1) [84] indicated a similar fold with the common catalytic triad located at identical sites on the enzyme surface, allowing for direct interaction with the PET polymers (Figure 3A), with the exception of Est30 (PDB ID: 1TQH) [85] (Figure 3B). Figure 3C illustrates the secondary structure of *Is*PETase—with the characteristic α-helix and β-strand fold architecture observed in all these enzymes—consisting of Ser (α4 helix), His (β8-α6 loop), and Asp (β7-α5 loop). The sequence alignment highlights a high degree of similarity in the secondary structure elements near the catalytic triad (Figure 3D), particularly those which contribute to the hydrophobicity of the catalytic cleft. Upon closer evaluation of the local environment near the catalytic triad, it is observed that it contains small and medium-sized hydrophobic residues including Ala, Ile, Leu, and Val. These residues are highly conserved and facilitate substrate binding mainly via nonpolar interactions with the PET backbone. Conservation of both the catalytic fold and active-site hydrophobicity in the enzymes, as shown in the present study, suggests strong evolutionary constraints to maintain effective PET-binding and catalysis. The implementation of MD simulations also provides additional support for the roles of loop mobility, active-site hydration, and conformational dynamics in the control of access for PET to the catalytic groove and in the stabilization of key reaction intermediates. These results provide a structural and mechanistic foundation to interpret the effects of site-directed mutations on the functioning of the enzymes, as described in the following sections.

### 3.4. Insights into the Role of Mutations from MD Simulations

Site-directed mutagenesis is one of the most common bioengineering techniques used to modify enzymes [88]. The method inserts point mutations on specific areas of the enzyme with the aim of changing specific functions. Mutations can either directly affect protein activity or promote enzyme stability through improvements in thermostability. By introducing targeted point mutations at specific residues, researchers can affect various functional properties of the enzyme, such as substrate binding, catalytic turnover, or overall stability. Depending on their location and nature, these mutations can directly influence catalytic performance through alteration of the active site environment or can confer increased structural rigidity, loop stability, or improved resistance to industrial conditions. While experiments can provide quantitative data on degradation, they cannot offer sufficient information regarding the underlying mechanism(s). In this context, MD simulations allow for the exploration of these mechanisms by providing an atomistic-level view of the interactions between the enzyme and the plastic polymer over time.

Although classical MD simulations are not capable of modelling bond breaking events and thus are limited to the modeling of the overall catalytic pathway [40], they provide critical information on mechanistically relevant properties. These properties include substrate binding orientation, flexibility of catalytic loops, accessibility of the active site, and stabilization of hydrogen bonding or hydrophobic interactions between the enzyme and the ligand. Throughout the reviewed studies, MD simulations were used to characterize the structural effects of the mutations and their relationship to experimentally characterized changes in PET degradation efficiency. For the majority of cases, enhanced flexibility of the loop structures proximal to the active site was reported in mutants with increased catalytic activity [32,57,60,62,70,73]. Additionally, analysis of Root Mean Square Fluctuations (RMSF) [32,57,60,62,64,68,69,70,75], hydrogen bonding [59,61,69,71], and significant residue–substrate interactions [32,58,59,63,65,66,73,74] provide quantitative descriptors that help to explain the functional implications of mutagenesis. Table 2 summarizes the mutations investigated in the reported studies, along with the experimental results and their effects on PET degradation, while Table 3 summarizes the results from MD simulations (with further details given in Extended Table S3). The effects of the mutations for each mutant, discussed in the text, regarding catalytic activity and the thermostability are summarized in Table 4.

#### 3.4.1. Improvement of Catalytic Activity

Enhancing the catalytic efficiency of PET-degrading enzymes can be achieved by focusing on the particular structural and dynamic features responsible for substrate binding and catalysis. Mutations that increase the hydrophobicity of the binding pocket, modulate loop flexibility, or strengthen specific non-covalent interactions between the enzyme and the PET polymer [89] have been shown to significantly improve catalytic activity. Among the reviewed studies, various strategies have been employed to shed light on these mechanisms. MD simulations can identify key catalytic aspects by modelling the complexes in various states and conditions. For example, increased RMSF values in loop regions within the binding site are indicative of greater flexibility in the specific areas, which facilitates substrate accommodation [35]. In addition, mutation of areas in the protein to reduce the distance between the catalytic triad and the attached PET polymer can promote the formation of significant interactions, i.e., hydrogen bonds and π-π stacking [90], thereby lowering the energetic barrier for catalysis. Finally, binding free energy calculations, particularly those performed using molecular mechanics with continuum solvent models for binding free energy estimation methods, can be used to identify key residues involved in enzyme–substrate interactions by estimating their contributions to the overall binding affinity [69].

A. Interactions between aromatic rings

The introduction of aromatic residues, such as Trp and Tyr, in the vicinity of the active site has been shown to significantly enhance enzyme activity [32,57,61,62,67]. This effect is potentially instigated by the π-π stacking interactions with the substrate, which may stabilize it inside the cavity region. The mutation His218Ser in the loop that contains the catalytic Asp (β7-α5 loop, Figure 3C) disrupts the interaction between Trp190 (β6–β7 loop, Figure 3C) and His218 in BhrPETase. In the double mutant BhrPETase_M2, Trp190 rotates more freely leading to a π-π stacking interaction with the PET polymer and to a 165% enhancement in product release over BhrPETase_WT [32]. Notably, Zheng et al. [61] employed a reverse approach in the LCC_ICCG enzyme, where Trp190 flexibility was intentionally reduced via the His218Tyr mutation leading to increased activity. The mutation provided room for π–π stacking between Tyr218 and Trp190, thereby facilitating a T-shaped interaction between Trp190 and the PET substrate. The replacement resulted in an increase in catalytic activity by 27% compared to LCC_ICCG at 72 °C. Consequently, the hydrogen bond count between LCC_ICCG and PET increased from 2.3 to 3.8 and 4.8 in the double and triple mutants LCC-A2 and LCC-A3, respectively [61]. Zheng et al. [62] utilized the same mutation (notated as His183Tyr) to design the one site mutation variant LCC_ICCG_Μ1-H183Υ, achieving a 69.4% increase in product release compared to LCC_ICCG at 70 °C. In this case, the hydroxyl group of Tyr forms a hydrogen bond with the N–H group of Trp190 (notated as Trp155), further influencing the side-chain flexibility of Trp [62]. Therefore, improvements in catalytic efficiency can be achieved either by restricting or allowing the movement of Trp, depending on the type of the mutated residue. Less bulky residues (e.g., Ser), for instance, allow for increased mobility. Inducing the π-stacking interaction between Tyr and the PET aromatic ring was also achieved by Sevilla et al. [67] in *Is*PETase through the Ser238Tyr mutation in the loop neighboring the catalytic His (β8-α6 loop, Figure 3C). The binding energy, computed by Molecular Mechanics/Generalized Born Surface Area (MM-GBSA) calculations, decreased by 20% (from −21.2 kcal/mol for *Is*PETase_WT to −25.5 kcal/mol for *Is*PETase_S238A), showing a 3.3-fold increase in activity for the mutant compared to the *Is*PETase_WT [67]. In another study, Lu et al. achieved a roughly 10% increase in PET depolymerization with the Leu91Trp (β4-α3 loop, Figure 3C) mutation compared to the triple mutant Est1_MPP, enhancing the stabilization of the substrate thought π-π stacking [57]. Collectively, these findings highlight the mechanistic importance of introducing aromatic residues such as Trp and Tyr in close proximity to the active site, particularly within the structurally flexible loops flanking the catalytic triad. MD simulations confirm that both the increased and restricted motion of key residues, such as Trp190, can be beneficial depending on the local structural context and the type of stacking interaction formed. Mutations in loop regions, especially those adjacent to the catalytic Asp, emerge as powerful levers for modulating the dynamics and electrostatic environment of the catalytic pocket, thereby significantly enhancing PET degradation efficiency.

B. Modification of the hydrophobic pocket

Another effective approach to enhancing the catalytic activity of PET-degrading enzymes is to redesign the active site by increasing its hydrophobicity or expanding the substrate-binding cavity. These adjustments are considered as a means to enhance substrate accessibility and orientation, particularly for hydrophobic polymers such as PET. MD simulations have been considered instrumental in demonstrating how these mutations impact local dynamics and molecular interactions that may not be easily realized from static structural models. Lu et al. [57] reported that the five site mutant Est_5M exhibits higher RMSF values at the two mutation sites close to the binding cavity. The introduction of the hydrophobic amino acids Ala and Met at positions 93 (β4-α3 loop, Figure 3C) and 213 (β8-α6 loop, Figure 3C) increased the hydrophobic surface area in the substrate-binding region. Substituting the Gln at position 93 with Ala introduced a smaller, nonpolar side chain that effectively widened the cavity and reduced electrostatic repulsion, allowing the PET substrate to access the catalytic Ser more efficiently. Additionally, the introduction of Met at position 213 increased the hydrophobic surface area, which potentially facilitates PET hydrolysis. Specifically, the Asn213Met mutation increased the depolymerization percentage from 1% in Est1_WT to approximately 22% in Est1_N213M [57]. Replacing hydrophilic with hydrophobic residues reduces the interaction with water (the solvent), facilitating the approach of the hydrophobic substrate towards the active site. Ding et al. [60] reported that the Glu173Gln mutation, at the loop of catalytic Asp (β7-α5 loop, Figure 3C), significantly increased the atomic fluctuations at the mutation site. This is likely due to the disruption of the electrostatic repulsion between Glu173 and catalytic Asp by a non-charged residue, allowing greater flexibility of the substrate-binding pocket in the six site mutation LCCICCG_I6M, leading to an approximately 40% increase in product yield compared to LCC_ICCG at 75 °C [60]. Zheng et al. [61] reported that the substitutions Asn248Asp and Ser247Ala (β8-α6 loop, Figure 3C) in LCC_ICCG broadened the narrow channel at the PET-binding region between positions 247 and Ile243, with structural analysis showing an increase in channel width from 5.9 Å in the LCC_ICCG to 8.5 Å and 8.9 Å, in the two and three site mutation variants LCC-A2 and LCC-A3, respectively. Notably, LCC-A2 showed a 23% increase in activity compared to the single mutant (His218Tyr). However, the addition of Ser247Ala did not lead to any further improvement in activity when tested experimentally [61]. This observation suggests that the increased size of the cavity allows the PET monomers to reach the active site more effectively, allowing for better substrate binding and higher catalytic efficiency. Introduction of new mutations does not, however, always make an additive contribution to the enzyme’s catalytic activity. Chen et al. [73] replaced the branched amino acid Ile213 (β8-α6 loop, Figure 3C) with Lys in TfCut2_WT. This specific change not only expanded the hydrophobic binding pocket but, more importantly, provided a long-range electrostatic effect that allowed the PET molecule to be correctly positioned closer to the active site. As a result, the degradation rate increased by 13% with the addition of the Ile213Lys mutation compared to the triple mutant variant TfCut2_M3a [73]. As before, the MD simulations demonstrated that the catalytic site loops are important for substrate orientation inside the binding site. The introduction of small hydrophobic residues (e.g., Ala and Ile) into these loops leads to expansion of the active site cavity and better accommodation of the substrate.

C. The Role of Glycine (Gly) and Phenylalanine (Phe) Substitutions in Catalytic Loops

The introduction of Gly in the catalytic loop regions has emerged as a promising approach for enzymatic activity enhancement by increasing local flexibility and expanding the substrate-binding pocket. Gly, due to its minimal side-chain steric bulk, permits greater backbone mobility compared to other amino acids, thereby altering the dynamic behavior of loop segments critical for substrate access and orientation. Zheng et al. [62] reported that the Leu124Gly mutation in LCC_ICCG increased flexibility (higher RMSF values) of the β5 strand, reducing steric hindrance in the active site cleft. Since this residue is adjacent to the catalytic Ser (α4 helix, Figure 3C), it is hypothesized to indirectly affect the flexibility of the β4 strand and β4–α3 loop (residues 114–127), enhancing substrate positioning. The variant LCC_ICCG_M2-L124G demonstrated an 11% enhancement in PET degradation at 70 °C compared to the single mutant LCC_ICCG_Μ1-H183Υ. The mutation maintains the hydrogen bond with Lys147 located on the β6 sheet, while modifying the interaction with surrounding residues, increasing flexibility without disrupting the enzyme structure [62]. Additionally, in the study of *Pp*PETase [70], the triple mutation Tyr239Arg/Phe244Gly/Tyr250Gly, forming the *Pp*PETase_M3 mutant, in the catalytic loop of His (β8-α6 loop, Figure 3C) caused the widening of the substrate-binding channel, allowing easier access of long-chain PET to the active site. The flexibility conferred by the less bulky Gly residues is further enhanced by the polar nature of Arg that may lead to increased interactions with the substrate. MD simulations showed elevated RMSF values within this loop, confirming enhanced flexibility [70]. The proposed MD models highlight the importance of binding site flexibility which is accompanied by a 3.1-fold increase in the concentration of degraded products in PET powder degradation experiments [70]. Therefore, the computational studies showed that mutations introducing less bulky amino acids, such as Gly, into the catalytic loop enhance the mobility of the residues in the active site cleft as indicated by increased RMSF values and it influencing the width of the binding channel.

In the loop containing the catalytic His, Phe is often replaced with other hydrophobic residues, such as Ile. Ile has a flexible and less bulky side chain allowing for better positioning of the substrate inside the active site. Additionally, Ile may alleviate steric hindrances caused by Phe and potentially strengthen enzyme–substrate interaction [59,73]. In Tournier et al. [59], the Phe243Ile mutation led to a 69% enhancement in activity in the double mutant LCC_M2. Particularly, the distance between the catalytic residues Ser and His was measured at 2.8 Å, with hydrogen bonds forming 90% of the simulation time in the four site mutations LCC_ICCG. In contrast, in LCC_WT the mean distance was approximately 4 Å, with hydrogen bonds forming only 15.2% of the time [59]. The increased hydrogen bond interactions between His and Ser may lead to increased interactions with PET upon substrate binding and thus improve catalytic efficiency. A similar strategy was employed by Chen et al. [73] for TfCut2, where the degradation rate increased from 3% in the TfCut2_WT to 30% by importing the mutations His184Ser and Phe209Ile (TfCut2_M2a). In TfCut2_WT, Phe209 and Ile213 form together a hydrophobic cluster, creating a narrow pocket that is too small to fully accommodate the PET substrate. Additionally, the π-π interactions between Phe209 and the PET benzene ring pulls the PET chain outward, probably impeding its correct orientation in the catalytic site. By mutating Phe209 to Ile and Ile213 to Lys, the pocket becomes more spacious, allowing better accommodation of the PET molecule and enhancing degradation efficiency [73].

Interestingly, Cui et al. [32] reduced the hydrophobicity of the active site cleft by introducing the Phe243Thr mutation into the six site mutant BhrPETase_M6. The substitution reduced steric hindrance and increased active site flexibility, as shown by the higher RMSF values compared to BhrPETase_WT. The enhanced substrate binding within the active site cleft improved catalytic efficiency since the distance between the catalytic Ser and PET is reduced from 4.9 Å to 4.2 Å [32]. When combined with the Trp104Leu mutation (α2 helix, Figure 3C), which disrupts the interaction with Pro248 (α6 helix, Figure 3C), product release from the eight mutant TurboPETase was increased by 71% compared to six mutant BhrPETase_M6 [32]. Moreover, mutation with Leu, as reported by Zhang et al. [58], also improves enzymatic activity. By replacing Gly130 with Leu in the Est30_M8 double mutant, the hydrophobic interaction between Leu130 and the benzene ring of the substrate is enhanced. As a result, catalytic efficiency increases from a 5.1-fold improvement with the single mutant Est30_M2 to a 36-fold increase with the Est30_M8 double mutant. Additionally, the frequency of hydrogen bond formation between the catalytic Ser and the substrate increased by approximately 120% (from 24.9% to 55.0%) [58]. Similar to the information reported in the previous section, the replacement of Phe with smaller hydrophobic residues (e.g., Ile) in the loop containing the catalytic His improves substrate positioning in the active site. This observation is supported by shorter catalytic distances and the formation of hydrogen bonds between the enzyme and the substrate, as reported above.

Collectively, these data establish that mutations in the loop containing the catalytic His—particularly those for Gly or Phe residues—are responsible for modulating substrate binding and active site structure. Reducing steric repulsion or establishing novel electrostatic and hydrophobic contacts maximizes positioning of PET within the catalytic cleft so that catalytic distances are diminished, hydrogen bonding is increased, and turnover is augmented, thereby enhancing the rate and degree of PET degradation. These trends are invariably substantiated with MD-derived structural and dynamic descriptors, emphasizing the predictive power of computational approaches in enzyme engineering.

#### 3.4.2. Thermostability Improvement

Improving enzyme thermostability remains an important goal in plastic biodegradation engineering. Enzyme flexibility analysis by MD, complemented by the rational introduction of structure-stabilizing residues and interactions, offers a comprehensive strategy for designing stable enzymes that can operate under the heat conditions required for large-scale PET recycling processes. As reported in Table 2, most of the studies were conducted at high temperatures (>40 °C), which comprise the industry standard. At these temperatures, the catalytic efficiency of enzymes involved in PET degradation is higher due to the high glass transition temperature of PET (~70 °C), above which the plastic becomes less viscous with higher mobility of its polymer chains [91]. Experimentally, enzyme thermostability is commonly quantified by parameters such as the melting temperature (*T_m_*) or half-inactivation temperatures after 60 min of incubation (*T*_50_^60^). Thermostability is indirectly quantified by MD simulations via analyses such as RMSF, with lower values indicating structural stiffness and higher stability, especially in the vicinity of the catalytic groove [57]. Unlike catalytic activity, where active site flexibility is desirable, decreased loop flexibility away from the groove has been associated with enhanced protein stability. It is interesting to observe that the flexibility requirements for thermostability differ considerably from those associated with catalytic activity [92]. Loop flexibility localized close to the active site enables substrate binding and turnover, but increased peripheral loop mobility can potentially lead to destabilization of the overall protein structure at elevated temperatures. Therefore, limiting the flexibility of non-catalytic loops, α-helices, and β-sheets without sacrificing enzyme conformational plasticity within the active-site environment remains a challenging task in thermostability engineering. To this end, disulfide bridges formed between cysteine residues have been employed as means to introduce tertiary structure stabilization by reducing conformational entropy [93]. Similarly, salt bridges and long-range electrostatic interactions improve thermal resilience by stabilizing domain architecture [93]. In addition, the presence of hydrophobic residues (e.g., Tyr, Phe, Ile, Ala, Val, and Leu), along with Pro [94], in the interior of proteins or surface loops reduces the exposure to solvent and increases the hydrophobic effect, thereby further stabilizing the folded structure. These experimental observations are supported by the MD simulations reported in the included studies (Table 2 and Table 3). Since PETase variants of enhanced thermostability are frequently characterized by reduced RMSF values in secondary structure elements and solvent-exposed loops, they are often accompanied by richer hydrogen bonding networks and improved packing density. When this information is combined with experimental validation (e.g., *T_m_* or residual activity assays), these computational metrics provide a reliable framework for predicting and optimizing thermal stability in engineered PET hydrolases.

A. Formation of disulfide bonds

Engineering of disulfide bonds is a powerful means to enhance PET hydrolase thermostability. However, the precise positioning of the engineered bonds is critical. To this end, MD-derived flexibility profiles and structural mapping can yield thermostable enzyme variants retaining or even improving catalytic efficiency and warrant their use under industrial thermal conditions. In this regard, one of the more frequent approaches for increasing PET-degrading enzyme thermostability is the rational addition of disulfide bridges. These bridges are typically engineered between cysteine pairs to reduce regional or global conformational flexibility and, consequently, increase structural rigidity under thermal stress. Most importantly, disulfide bonds introduced away from the catalytic center can make a significant contribution to thermostability without compromising enzyme activity. Specifically, Cui et al. achieved an increase in *T_m_* by 2.0 °C in BhrPETase_M3 by inserting Cys residues in positions 251 and 281 [32]. Additional investigations identified two opposing effects associated with the incorporation of disulfide bonds near the active site, specifically within the loop containing the catalytic His. For example, Tournier et al. found that introducing disulfide bonds by mutating Asp238Cys (β8-α6 loop, Figure 3C) and Ser283Cys (β9 strand, Figure 3C) in LCC_ICCG increased the *T_m_* of LCC_WT by 9.8 °C but potentially reduced activity by 28% [59]. This was attributed to decreased flexibility in the catalytic His loop, potentially impeding substrate accommodation or product release [59]. In contrast, similar disulfide-engineering strategies yielded favorable outcomes in related PETases. Joho et al. using the same mutations at *Ps*PETase (notated as Asn233Cys and Ser282Cys) achieved an increase in *T_m_* of 11.4 °C with a 2.8-fold increase in the activity of PETase^ACC^ [71]. Increased activity was also reported by Han et al. who observed that the Ala212Cys/Thr249Cys double mutation (*Sc*PETase_M2) resulted in a more than 2.3-fold increase in hydrolytic activity toward PET at 50 °C compared to *Sc*PETase_WT [70]. In all cases, MD simulations were used to characterize the dynamic and structural effects of disulfide bond insertion. Decreased RMSF values in disulfide-constrained variants consistently displayed increased local rigidity, particularly in loop regions that were previously thermally destabilized. MD analyses also indicated that an optimized enhancement of local rigidity, particularly when localized to non-catalytic loops, could increase overall structural stability without diminishing, or even while augmenting, active-site flexibility. On the other hand, disulfide bridges that are too far from dynamic catalytic sites may impair activity by restricting the conformational movement required for substrate turnover.

B. Introduction of polar amino acids and the formation of salt bridges

Another effective strategy for enhancing thermostability is the introduction of salt bridges. Mutations that introduce negatively [66,74] or positively charged residues [64,66,68] on the surface of the protein have been demonstrated to enhance thermostability and stabilize flexible loops. This is particularly effective when charges are placed in solvent-exposed regions or at interdomain interfaces, where they can reduce conformational entropy and enhance electrostatic cohesion. For example, Meng et al. introduced two negatively charged residues in TfCut2, Leu32Glu (α2 helix, Figure 3C), and Ser113Glu (α3-β5 loop, Figure 3C) that formed an intramolecular salt bridge with the neighboring positive residues Arg31 and Arg116 [74]. The result was a 3-fold increase in hydrolysis activity towards crystalline PET powder at 65 °C. Similarly, introducing the Thr237Gln mutation into the double mutant TfCut2_M2b to form the TfCut2_M3b triple mutant led to a *T*_50_^60^ value of 70.5 °C, which was 5.7 °C higher than that of the TfCut2_WT, indicating a significant improvement in thermostability. The variant also exhibited an average binding free energy of −81.8 kJ/mol to PET, significantly lower than the −64.3 kJ/mol in the TfCut2_WT. The increased substrate binding affinity is further supported by the reduced distance between the catalytic Ser and the ester bond in PET, which decreased from 8.2 Å to 3.7 Å in the triple mutant TfCut2_M3b, highlighting the importance of this characteristic of the enzymatic process. This structural rearrangement resulted in a 5.3-fold increase in activity of the triple mutant TfCut2_M3b compared to TfCut2_WT [74]. Similarly, Qu et al. introduced the positively charged Arg and the negatively charged Glu in the Ile168 and Ser188 positions in *Is*PETase (*Is*PETase_M2f), respectively [68], which resulted in a *T_m_* increase by 8.7 °C. The respective MD simulations have shown that the introduction of these mutations led to lower RMSF values in the protein regions 165–175 (α4 helix) and 186–200 (β6-β7 loop, Figure 3C), an indication of increased rigidity. *Is*PETase_M2f showed a 4.3-fold increase in activity, while *Is*PETase_M2e (with the mutation Ser188 to Asp instead to Glu) showed a 3.8-fold increase compared to the *Is*PETase_WT at 40 °C. The superior performance of the Ile168Arg/Ser188Glu strain has been attributed to a stable salt bridge between Arg168, Asp186, and Glu188. This was highlighted by a distance of 4.5 Å between Arg168’s CZ and Glu188’s CD carbon atoms, as observed in the final 5ns of the MD simulations performed [68]. In contrast, the salt bridge in the Ile168Arg/Ser188Asp mutant is less stable, with the C-C distance calculated at 5.5 Å between Arg168 and Asp188. Therefore, it can be inferred that the longer side chain of Glu allows for the formation of a more stable salt bridge with Arg168, as deduced by MD simulations [68].

Additional electrostatic stabilization was achieved by introducing the Trp159His mutation in *Is*PETase (*Is*PETase_M1a), as reported by Meng et al. [64], where a salt bridge was created between Asp118 and Arg123 on the *Is*PETase surface (α3 helix, Figure 3C). The substitution of Trp by His led to the reduction of the N-O distance between Asp118 and Arg123 from 5.9 Å in the *Is*PETase_WT to 2.7 Å in *Is*PETase_M1a. As a result, the *T_m_* increased by 6.8 °C, while the catalytic efficiency showed a 1.4-fold increase at 40 °C [64]. Similarly, Yin et al. demonstrated that the introduction of two positively charged Arg and Lys residues improved both thermostability and activity of *Is*PETase [66]. Through the introduction of the Ile139Arg mutation (*Is*PETase_M1b) at the α4 helix (Figure 3C) a *T_m_* increase of 8.7 °C was achieved, while the Ser92Lys mutation (*Is*PETase_M1c) at the α3 helix (Figure 3C) led to a modest 1.7 °C increase; this was further enhanced by 3.7 °C when combined with the Arg251Ala mutation (*Is*PETase_M2c). MD simulations revealed that the distance between the –OH group of the catalytic Ser and PET decreased from 5.1 Å in the *Is*PETase_WT to 3.4 Å and 4.0 Å in the *Is*PETase_M2c and *Is*PETase_M1b variants, respectively. As a result, the degradation activity of *Is*PETase_M2c and *Is*PETase_M1b had a 2.9-fold and 3.6-fold increase at 40 °C, respectively. Both *Is*PETase_M2c and *Is*PETase_M1bvariants also performed well at 50 °C [66]. In another study, Qu et al. found that the mutations Asp186Asn (*Is*PETase_D186N) and Asp186His (*Is*PETase_D186H) led to an increase in *T_m_* by 8.9 °C and 8.8 °C, respectively [69]. The MD simulations revealed a reduction in RMSF values at 40 °C and 100 °C in the regions 181–197 (β6-β7 loop, Figure 3C) and 205–222 (β7-α5 loop and α5 helix, Figure 3C), where Asp186 is located. This stabilization enhances protein structural integrity, thereby improving the catalytic activity at high temperatures. This was further supported by Molecular Mechanics/Poisson-Boltzmann Surface Area (MM-PBSA) calculations, which showed a slight increase in the binding affinity for *Is*PETase_D186N (−93.2 kJ/mol) and *Is*PETase_D186H (−91 kJ/mol) compared to the *Is*PETase_WT (−88.2 kJ/mol). Moreover, the product yield at 40 °C displayed a 3.7- and 3.4-fold increase for *Is*PETase_D186N and *Is*PETase_D186H, respectively, compared to the *Is*PETase_WT. Notably, the Asp186His and Asp186Asn mutations also led to the formation of three stable hydrogen bonds in the β6-β7 loop (Figure 3C) involving Asn/His186 and the neighboring Ser187 and Ser188 [69].

However, there are cases where the introduction of charged amino acids may not improve overall stability or even has a detrimental effect, leading to protein destabilization. For example, in *Is*PETase, the Asp157Glu mutation (β6-β7 loop, Figure 3C), when combined with Ser92Lys (*Is*PETase_M2d) or Ile139Arg (*Is*PETase_M2b), decreased the *T_m_* by 4 °C and 9.1 °C, respectively, as demonstrated in the study of Yin et al. [66]. MD simulation analysis showcased that Glu157 disrupts the stabilizing interaction with Lys92, promoting solvent exposure and contributing to enzyme structure destabilization [66].

Additionally, Zhang et al. [58] reported that the introduction of Ile171Lys in Est30 (Est30_M2) had a negative impact on thermostability, resulting in a 9 °C decrease in *T_m_*. This decrease in stability is likely a trade-off, as the mutation facilitates hydrogen bond formation with the substrate, ultimately enhancing enzymatic activity [58]. MD simulations demonstrated a rise in the frequency of close contacts between the catalytic Ser and the substrate, from 11.7% in the Est30_WT to 24.9%. This may possibly account for the observed 5% increase in catalytic efficiency [58], as the PET moiety is positioned closer to the catalytic site (Figure 2A). Disruption of protein hydrophobicity was also achieved by introducing polar residues inside the active site; this resulted in a reduction of protein stability but enhanced substrate stabilization in the cavity via hydrogen bond interactions. Specifically, the Met127Ser mutation led to a decrease in *T_m_* by 4.4 °C in the triple mutant Est30_M14 triple mutant. Enzyme rigidity loss was counterbalanced by the introduction of polar groups that may enhance hydrogen bond formation inside the active site cavity. These interactions potentially contribute to the stabilization of the transition state during PET degradation and can explain the observed 96.3-fold increase in catalytic efficiency of the Est30_M14 mutant [58]. This shows that local polarity close to the active site can enhance the binding affinity of the substrate at the cost of structural rigidity.

Joho et al. [71] observed increased activity in the *Ps*PETase, Ser136Glu mutant (α3 helix, Figure 3C), which had a minimal impact on thermal stability but resulted in a 1.3-fold increase in hydrolytic activity compared to the PETase^ACC^ variant at 45 °C. Despite this improvement, no direct formation of a new salt bridge was detected. The authors proposed that the observed effect might stem from a transient intra-protein interaction or enhanced enzyme–solvent interactions [71]. MD simulations did not reveal significant differences between the strains, suggesting that the observed changes may not be directly attributed to the mutations.

Finally, the location of the salt bridge plays a crucial role in protein performance. The mutations may have a negative impact on activity if introduced inside the active site pocket, as they could disrupt the hydrophobic environment. Cui et al. demonstrated this by introducing positively charged residues, Lys and Arg and specifically the Asp238Lys mutation in the BhrPETase loop of the catalytic Asp and the Ala209Arg mutation in the loop of the catalytic His, which, despite increasing the *T_m_* of double mutant BhrPETase_M2 and the five site mutant BhrPETase_M5 by 8.5 °C and 1.5 °C, respectively, nonetheless led to a decrease in activity due to the rigidification of the active site [32]. In summary, charged residues, particularly in the β6–β7 loop, play an important role with regard to enzyme stabilization. Decreases in RMSF values signify improved thermostability, while MD simulations provide insight into salt bridge distances and their contribution to structure stability. Additionally, the inclusion of polar residues is characterized by the presence of an effect on catalytic activity through changes in enzyme–substrate distances and binding affinity calculations.

C. Hydrophobic residue mutations

The replacement of existing hydrophobic residues is another route to enhance enzyme thermostability. This is achieved by improving the packing density of the hydrophobic core or loop regions, reducing solvent exposure, and increasing resistance to heat-induced denaturation. These mutations are particularly effective in solvent-exposed loops or adjacent to β-sheets, which are more susceptible to heat-induced unfolding. For example, the smaller side chain of Gly reduces steric hindrance and enhances conformational flexibility, as demonstrated in several studies [58,59,64]. Tournier et al. substituted Tyr127 with Gly, which resulted in a 9.3 °C increase in the *T_m_* of the five mutant LCC_ICC variants, although this came at the cost of a 22% decrease in activity [59]. This highlights that such residue mutations can be a double edge sword by altering flexibility; even though Gly’s minimal steric effects stabilize protein structure under heat stress, it simultaneously disrupted critical substrate interactions required for efficient catalysis. In another example, Zheng et al. reported that the Gly130Leu mutation in Est30 increased *T_m_* by 2.2 °C, further supporting the role of substituting hydrophobic residues in improving thermal stability [58]. This substitution also improved catalytic activity when paired with additional mutations, as discussed in earlier sections.

Beyond simply replacing flexible residues, thermostability improvements have also been observed through hydrophobic-to-hydrophobic substitutions. Specifically, Meng et al. introduced the additional Phe229Tyr mutation to the *Is*PETase_M1a variant, which resulted in a *T_m_* increase by 3.6 °C compared to the *Is*PETase_WT [64]. While the single Phe229Tyr mutation did not greatly affect catalytic efficiency compared to the *Is*PETase_WT, in the Trp159His/Phe229Tyr double mutant (*Is*PETase_M2a) the activity showed a 1.4-fold increase [64]. The RMSF analysis showed decreased values in the protein region delimited by residues 205–235 between β7-α5 loop and β8 strand (Figure 3C), where the majority of the calculated additional hydrogen bonds formed as a result of the Phe229Tyr substitution [64]. These examples highlight how the introduction of a hydrophobic residue, either for augmented packing density or to facilitate novel local contacts, can achieve a significant enhancement in thermal stability. However, as with other methods of mutation, balance is required so as not to perturb active-site flexibility or substrate binding. MD simulations play a vital role in the evaluation of the structural and dynamic effects of these mutations, particularly through RMSF analysis and hydrogen bond mapping, which provide information on protein rigidity and interaction networks.

D. The role of Alanine (Ala) and Valine (Val)

The substitution of surface or loop residues with small, hydrophobic amino acids like Ala and Val has been employed extensively to enhance thermostability and catalytic activity of PET hydrolases. The specific residues have various roles such as stabilizing local loops, fine-tuning enzyme–substrate interactions, and optimizing catalytic geometry, especially in flexible structural regions close to the active site. Ding et al. investigated the effect of mutating two positively charged residues, namely Lys95 (α2 helix, Figure 3) and Arg280 (α7-β9 loop, Figure 3C) to Ala in V3 PETase [75]. This V3 PETase variant is a thermostable enzyme with mutations Gly119Gln, Asn233Cys, Ser238Trp, and Ser282Cys, which enhance stability through the insertion of an additional disulfide bond between Cys233 (β8-α6 loop, Figure 3C) and Cys282 (β9 strand, Figure 3C). The additional disulfide bridge, along with the increased stacking interactions between Trp238 and the terephthalate ring, lead to a 14 °C increase in *T_m_* compared to the *Is*PETase_WT. The introduction of the single Lys95Ala mutation (V3 PETase_M1) further increased the *T_m_* by 1 °C, while Arg280Ala combined with the double mutant V3 PETase_M2 resulted in a 3.8 °C increase compared to the V3 PETase_WT. RMSF analysis showed that the Lys95Ala mutation stabilized the active site, leading to a 1.4-fold reduction in PET film degradation activity. Interestingly, Lys95Ala increased activity by 1.3-fold on PET powder after 24 h. Combining Arg280Ala with the V3 PETase_M2 showed no additional activity improvement at 40 °C and only a slight activity increase at 50 °C (Table 4) [75].

Similarly, Yin et al. reported that the Arg251Ala mutation of the *Is*PETase_M1c variant resulted in a *T_m_* increase of 3.7 °C [66]. The substitution of the charged Arg251 with Ala at the α7-β9 loop (Figure 3C) is likely to stabilize the flexible loop region, enabling the enzyme to retain its catalytic activity at 50 °C, at which temperature the *Is*PETase_WT enzyme was inactivated [66]. Mutations to Ala have also been shown to stabilize flexible loop regions, particularly those near the active site, thereby improving the enzyme’s ability to accommodate the substrates. Joho et al. substituted Asp186 to Ala in *Ps*PETase (PETase^A^), leading to an increase in flexibility of the adjacent Trp185 side chain [71]. The Asp186Ala variant exhibited a 10 °C increase in *T_m_* while maintaining high catalytic activity, even at 50 °C [71]. Qu et al. observed an increase in *T_m_* of 11.8 °C and 12.9 °C for Asp186Ala (*Is*PETase_D186A) and Asp186Val (*Is*PETase_D186V) mutations of *Is*PETase, respectively [69]. MD simulation analysis revealed a decrease in RMSF values for regions 181–197 of the β6-β7 loop and 205–222 at the β7-α5 loop and a5 helix, (Figure 3C) where Asp186 is located. Notably, the key PET binding Trp185 and Ile208 residues located in the first and second regions, respectively, became more rigid, which in turn impacted the catalytic activity. This was further supported by MM-PBSA binding energy calculations, which showed a decrease in binding affinity for *Is*PETase_D186A (−84.8 kJ/mol) and *Is*PETase_D186V (−65.3 kJ/mol) compared to *Is*PETase_WT (−88.2 kJ/mol). However, at 40 °C, the PET degradation efficiency increased 2.5-fold and 3.6-fold for *Is*PETase_D186V and *Is*PETase_D186A, respectively. According to Qu et al., substituting Asp186 with hydrophobic residues (Ala or Val) stabilizes the π-alkyl interaction with Phe191, enhancing its loop stability [69]. However, not all substitutions of hydrophobic residues lead to improved outcomes.

Interestingly, Sevilla et al. reported that, although replacing Ile208 with Val in the loop containing the catalytic Asp resulted in a 20% increase in calculated binding energy for PET (−25.5 kcal/mol) compared to the *Is*PETase_WT (−21.2 kcal/mol), in vitro assays revealed a decrease in enzymatic activity [67]. This discrepancy was attributed to the fact that the Ile208Val mutation disrupted several non-covalent interactions between Ile208 and the catalytic His, leading to a shift of His closer to Ser, which facilitates its deprotonation. Consequently, the flexibility of the catalytic His increased, which ultimately reduced the enzyme’s turnover rate [67]. The same study also investigated the Asn212Ala mutation in *Is*PETase, reporting a calculated binding energy of −28.4 kcal/mol for the mutant, compared to −21.2 kcal/mol for the *Is*PETase_WT. This mutation led to a 1.4-fold increase in enzymatic activity. The structural effect of Asn212Ala is suggested to bring its adjacent helix closer to the protein core, influencing residues 204–210 (β7-α5 loop, Figure 3C) and positioning the catalytic Asp residue in a more favorable conformation for catalysis [67].

Guo et al. mutated Ser238 in the *Is*PETase (*Is*PETase_S238A) catalytic His loop with Ala, which did not result in any significant change in *T_m_* compared to *Is*PETase_WT but led to increased PET hydrolysis activity, with a 55% increase in product release compared to *Is*PETase_WT at 30 °C [65]. MD simulations showed that the catalytically favorable conformations for Ser238Ala –where the Ser-His and His-Asp heavy atom distances were less than 3.5 Å—reached 75.4%, compared to only 10.1% for the *Is*PETase_WT, which could possibly explain the increase in PET degradation in the *Is*PETase_S238A [65]. This mutation also reorientated Trp185, which is linked to PET stabilization within the cavity. Specifically, the Trp185 side chain was positioned almost perpendicular relative to Tyr87 in the *Is*PETase_S238A variant, whereas it assumes a flat orientation in the *Is*PETase_WT that was also observed in the *Is*PETase_Y87E strain. Although the *T_m_* of *Is*PETase_Y87E increased by 12 °C relative to *Is*PETase_WT, it presented diminished activity due to the decreased formation of hydrogen bonds among the catalytic residues. This disruption of interactions led to a drop in the catalytically favorable conformations in *Is*PETase_Y87E to 12.8% [65].

A combination of Ala and Thr mutations has also been reported to enhance enzymatic activity. Joho et al. introduced a Ser214Thr mutation to the Asp186Ala (α5 helix, Figure 3C) variant of *Ps*PETase, which led to 1.1-fold increase in the enzymatic activity of PETase^ACC^ [71]. This increase was attributed to the disruption of the hydrogen bond between residue Thr214 and Pro184, enhancing the flexibility of the β6-β7 loop (Figure 3C) and the binding site. The median distance between the backbone hydrogen and oxygen atoms of Thr214 and Pro184 was found to be greater than that of Ser214-Pro184, which likely contributed to the increased activity of this variant [71].

Weigert et al. investigated the β3-α2 loop mutations, Val91Thr and Ser92Ala in PET6 (PET6-VSTA), located near the active site (Figure 3C) [63]. These mutations resulted in a 63% increase in product release compared to the PET6_WT in the presence of 1 mM NaCl. MD simulations revealed that the hydroxyl group of Thr91 frequently interacts with the π-system of the terephthalate units of the PET tetramer, forming potential OH–π interactions at a median distance of 3.5 Å. Additionally, a hydrophobic contact between Ala92 and PET was observed. As a result, the PET6-VSTA exhibited a significantly higher contact frequency between the ligand and the catalytic His (64%) compared to PET6_WT (18%) [63].

These findings collectively highlight the functional importance of Ala and Val for loop stabilization, active site preorganization, and catalytic interaction optimization. Enhancements in performance are consistently supported by MD-derived metrics like reduced catalytic distances, lower RMSF values, increased hydrogen bond occupancy, and increased contact frequencies between the enzyme and PET.

E. The role of Proline (Pro)

The introduction of Pro residues at specific PET hydrolase structural regions [95] has become an established strategy for improving thermostability, particularly when implemented in the loop, helix, and turn regions [96] away from the catalytic center. Pro’s structure imposes conformational rigidity on the protein backbone by limiting local flexibility and contributes to the overall stabilization of secondary structural elements [57]. Choosing the appropriate mutation site at which to insert a Pro residue is crucial, as the introduction of a kink in the backbone enhances thermostability and stabilizes helices and turns [94].

In this regard, Lu et al. demonstrated that the Ser115Pro mutation in the α3-β5 loop (Figure 3C) of Est1, which is located away from the active site, enhanced thermostability as evidenced by the lower RMSF values in this region [57], which is supportive of the hypothesis that insertion of Pro residues at solvent-exposed loops improves the structural rigidity of the enzyme. In contrast, introduction of mutations closer to the active site favors enzymatic activity rather than structural stabilization. This was showcased by the Thr215Pro mutation in the β8-α6 loop (Figure 3C), which resulted in an increase in PET depolymerization of 36% [57]. This suggests that Pro positioning acts as a fine-tuning mechanism for balancing catalytic performance with enzyme flexibility.

In another study by Ding et al., a similar pattern was reported upon the mutation of Asn213 and Thr157 to Pro in the β8-α6 and β6-β7 loops, respectively, (Figure 3C), which led to a 1.0 °C increase to the *T_m_* of LCCICCG_I6M compared to LCC_ICCG [60]. MD simulations further illustrated that Asn213Pro is responsible for a reduction in RMSF values near the active site, thereby illustrating that the introduction of Pro potentially enhances the stability of the active site by influencing ligand orientation, which in turn improves substrate degradation. Notably, the Thr157Pro mutation led to a smaller side chain and an enlarged substrate entrance, delimited by the Pro157 and Gln182 residues, which expanded by 0.3 Å, representing an approximate 60% increase compared to LCC_ICCG at 75 °C [60], which enhances substrate accessibility.

In summary, these findings underscore the dual role of Pro, which largely depends on its placement within the enzyme. When positioned away from the catalytic core, it contributes to loop stabilization, thereby enhancing enzyme thermostability. When placed close to the active site, it enhances catalytic activity by altering the conformation of flexible loops, widening the substrate entrance or enabling a more favorable substrate binding. MD simulation derived RMSF profiles coupled to experimental enzyme activity measurements confirm this modulating role in optimally designing PET-degrading enzymes for industrial applications.

F. The role of ions

The majority of the research studies reported here focused primarily on rational mutagenesis for improving catalytic efficiency as well as thermal stability, but a few of them explored the effects of ionic concentration and specific ions on enzyme stability and activity. As summarized in Table 5, only two studies have systematically investigated the impact of monovalent and divalent ions on PET hydrolase performance [63,72]. Weigert et al. evaluated the thermal stability of *Is*PETase and PET6 at varying NaCl concentrations [63]. At 50mM NaCl, *T_m_* values of *Is*PETase and PET6 were measured at 46.2 °C and 49.8 °C, respectively. Upon increasing NaCl concentration to 1M, the *T_m_* reached 52.7 °C and 57.7 °C for *Is*PETase and PET6, respectively; ref. [63] suggested a clear stabilizing effect on enzyme structure as a function of ionic strength. However, the opposite trend was reported regarding the catalytic performance of these two enzymes at elevated temperatures, as PET6 outperformed *Is*PETase at 50 °C, releasing 1.1μM of hydrolysis product compared to 0.9 μM for *Is*PETase. This demonstrates the promising PET degradation potential of PET6 under increased salt concentrations, suggesting that other PET hydrolases could follow similar trends under similar conditions [63].

Then et al. investigated the influence of introducing divalent cations such as Ca^2+^ or Mg^2+^ on the thermostability and activity of TfCut2 [72]. Addition of these cations resulted in an increase in TfCut2’s *T_m_* between 10.8 °C and 14.1 °C compared to the TfCut2_WT. At 65 °C, PET degradation—determined using semi-crystalline PET films’ weight loss after incubation with the enzymes—reached 12.6% in the presence of 10 mM Ca^2+^ and approximately 7% with 10 mM Mg^2+^, while it was negligible for the wild-type control enzyme [72]. The highest calculated probability densities for Ca^2+^ binding within a 3.7 Å radius of TfCut2 residues was observed for Glu253 (83.6%), Asp174 (81.2%), Asp204 (64.8%), and Gly205 (29.0%). In contrast, the corresponding probability densities for Mg^2+^ were significantly lower and involved Glu253 (18.7%) and Asp174 (9.6%). Residues Asp204 and Gly205 are located on the same loop as catalytic His, while Asp174 is proximal to the catalytic Asp, suggesting a correlation between cation binding and improved enzyme activity at higher temperatures, while Glu253 located at the C-terminal has no significant effect on activity [72].

These findings suggest that unique interactions between ions and proteins, particularly those between divalent cations and catalytically adjacent acidic residues, could have a stabilizing role and potentially an activating function in PET hydrolases. The variability in activity responses between enzymes subjected to the same ionic conditions also emphasizes the protein-specific properties of these interactions. As discussed previously, most studies have focused on the impact of mutations rather than exploring the impact of ionic concentration, indicating a significant research gap on this important parameter that affects enzyme stability and activity. Given that the current evidence suggests enhanced enzyme stability in the presence of increased Ca^2+^ or Mg^2+^ concentrations, further research is needed to systematically explore the diversity of ionic species—including monovalent, divalent, and other charged ions—and their concentration-dependent effects on enzyme structure and catalytic activity.

## 4. Limitations and Future Work

This review provides important insights into the application of MD simulations in enzyme engineering for PET degradation. However, several limitations should be acknowledged, as they may impact the interpretation and the broader applicability of the findings. A key limitation lies in the design of the search strategy, which involves five specific search terms to narrow down the number of retrieved studies. While this approach was effective in focusing the analysis, it may have excluded relevant studies that used alternative terminology or reported findings in different contexts. Consequently, the comprehensiveness of this review might be limited, potentially affecting the breadth of available evidence captured. Additionally, this review is primarily descriptive rather than quantitative. Due to the wide variation in experimental conditions and setups, protein systems, and types of analyses (including differences in kinetic assays, degradation properties, and MD simulation parameters), direct comparisons are not feasible and may lead to misinterpretations. A meta-analysis or quantitative synthesis would require a more focused subset of studies examining specific properties under standardized conditions.

MD simulations can be pivotal in the elucidation of the role of specific mutations—such as the introduction of disulfide bridges, aromatic/hydrophobic amino acids, and salt bridges—on both thermal stability and catalytic efficiency. However, due to the limitations of MD tools, it is important to adhere to strict simulation protocols to ensure more accurate and effective enzyme engineering outputs. MD simulations depend largely on the quality of the structural models, the type of force fields [97], and the biophysical relevance of the simulation conditions. Therefore, the heterogeneity in experimental parameters, like changes in pH, buffer composition, PET crystallinity, and temperature, can further limit direct comparisons within and between computational models. Moreover, structural ambiguities in the crystallographic or cryo-EM data of enzymes and their mutants, along with heterogeneity in the reported kinetic estimates, may introduce uncertainty in the process of calibrating the simulations against experimental benchmarks [98,99,100]. Future studies should consider systematically investigating the effects of different MD simulation parameters on the predicted structural and dynamic behavior of PET-degrading enzymes. To enable meaningful comparisons, it would be beneficial to analyze MD simulations conducted under consistent conditions and applied to the same enzyme system. Such efforts could help identify optimal simulation setups and contribute to more reproducible and predictive MD-based enzyme engineering strategies.

## 5. Conclusions

This systematic review aims to highlight the importance of computational methods, particularly MD simulations, for advancing our mechanistic understanding of enzymatic PET degradation. Even though experimental methods remain essential for the quantification of enzyme function and the confirmation of plastic degradation through product analysis or structure imaging techniques, they often fail to capture the mechanisms controlling enzyme–substrate interactions, mutational effects, and key conformational intermediates along the catalytic pathway at atomistic detail. In contrast, MD simulations enable the generation of time-resolved, atomistic insights into enzyme conformational motion, loop motion, substrate binding modes, and thermostability—factors that are essential for the rational design of enzyme engineering. The integration of computational simulations and experimental methods has revealed substantial synergies. MD analysis metrics, such as RMSF, binding free energies, and hydrogen bonding interactions, have not only been used to analyze and interpret experimental data, but also to guide the design of variants aimed at optimizing enzyme efficiency and resulting PET depolymerization. In particular, this systematic review highlights how MD simulations have helped advance the development of PET hydrolases with enhanced catalytic activity, substrate accessibility, and thermostability through targeting critical residues in functional loops, active site grooves, and structurally dynamic domains.

Among the enzymes investigated, *Is*PETase’s high catalytic efficiency under mesophilic conditions (i.e., 20–45 °C) and its structural adaptability to mutations render it an attractive candidate for large-scale biocatalysis [101]. Nonetheless, LCC, PET6, and TfCut2 present noteworthy alternatives in achieving thermostability and structural resilience at high temperatures, conforming with the operational need for handling PET near its glass transition temperature [60].

Future MD-guided enzyme bioengineering efforts should further pursue computational and experimental protocol harmonization. The simulation conditions need to model real-world industrial scenarios more realistically, including the diversity in polymers, temperature gradients, and interactions with the solvent. Conversely, the experimental protocols should incorporate mechanistically informed mutation designs derived from computational predictions, rather than purely making use of high-throughput screening techniques or empirical screening methods. Overall, this review demonstrates the prospects and strengths of MD simulations to act not merely as analysis tools but as active platforms for the computational design of enhanced PET-degrading enzymes. When meticulously coupled to experimental verification, they comprise an efficient, scalable, and mechanistically informed framework for engineering biocatalysts (enzymes) with improved efficiency and resilience. As the need to eliminate plastic waste is urgently growing, such synergies between computational and experimental approaches will become imperative for accelerating the translation of enzyme degradation technologies from the laboratory towards practical implementation at industrial and environmental scales.

## Figures and Tables

**Table 1:** Articles grouped by the type of enzyme studied.

Enzyme	Organism	Mutation	Short-Name	Ref.
BhrPETase	*Bacterium* HR29	-	BhrPETase_WT	[32]
		His218Ser/Phe222Ile	BhrPETase_M2	
		BhrPETase_M2/Asp238Lys	BhrPETase_M3	
		BhrPETase_M3/Ala251Cys/Ala281Cys	BhrPETase_M5	
		BhrPETase_M5/Ala209Arg	BhrPETase_M6	
		BhrPETase_M6/Trp104Leu/Phe243Thr	TurboPETase	
Est1	*Thermobifida alba* AHK119	-	Est1_WT	[57]
		Asn213Met	Est1_N213M	
		Est1_N213M/Thr215Pro	Est1_N213M/T215P	
		Est1_N213M/T215P/Ser115Pro	Est1_MPP	
		Est1_MPP/Gln93Ala/Leu91Trp	Est1_5M	
Est30	*Geobacillus* *stearothermophilus*	-	Est30_WT	[58]
		Gly130Leu	Est30_M1	
		Ile171Lys	Est30_M2	
		Est30_M2/Gly130Leu	Est30_M8	
		Est30_M8/Met127Ser	Est30_M14	
LCC	Metagenomic	-	LCC_WT	[59]
		Phe243Ile	LCC_Μ1	
		Asp238Cys/Ser283Cys	LCC_M2	
		LCC_Μ1/Asp238Cys/Ser283Cys	LCC_ICC	
		LCC_ICC/Tyr127Gly	LCC_ICCG	
		LCC_ICCG/Ser32Leu/Asp18Thr/Ser98Arg/ Thr157Pro/Glu173Gln/Asn213Pro	LCCICCG_I6M	[60]
		LCC_ICCG/His218Tyr	LCC_ICCG_Μ1-H218Υ	[61]
		LCC_ICCG_Μ1-H218Y/Asn248Asp	LCC-A2	
		LCC-A2/Ser247Ala	LCC-A3	
		LCC_ICCG/His183Tyr	LCC_ICCG_Μ1-H183Υ	[62]
		LCC_ICCG_Μ1-H183Υ/Leu124Gly	LCC_ICCG_M2-L124G	
		LCC_ICCG_M2-L124G/Ser29Ala	LCC-YGA	
PET6	*Vibrio gazogenes*	-	PET6_WT	[63]
		Val91Thr/Ser92Ala	PET6-VSTA	
*Is*PETase	*Ideonella sakaiensis* 201-F6	-	*Is*PETase_WT	[64]
		Trp159His	*Is*PETase_M1a	
		Phe229Tyr	*Is*PETase_F229Y	
		*Is*PETase_M1a/Phe229Tyr	*Is*PETase_M2a	
		Ser238Ala	*Is*PETase_S238A	[65]
		Tyr87Glu	*Is*PETase_Y87E	
		Ile139Arg	*Is*PETase_M1b	[66]
		*Is*PETase_M1b/Asp157Glu	*Is*PETase_M2b	
		Ser92Lys	*Is*PETase_M1c	
		*Is*PETase_M1c/Arg251Ala	*Is*PETase_M2c	
		*Is*PETase_M1c/Asp157Glu	*Is*PETase_M2d	
		Ile208Val	*Is*PETase_I208V	[67]
		Ser238Tyr Asn212Ala	*Is*PETase_S238Y *Is*PETase_S238Y	
		Ile168Arg/Ser188Asp	*Is*PETase_M2e	[68]
		Ile168Arg/Ser188Glu	*Is*PETase_M2f	
		Asp186Val	*Is*PETase_D186V	[69]
		Asp186Ala	*Is*PETase_D186A	
		Asp186Asn	*Is*PETase_D186N	
		Asp186His	*Is*PETase_D186H	
*Pp*PETase	*Pseudomonas paralcaligenes* MRCP1333	-	*Pp*PETase_WT	[70]
		Tyr239Arg	*Pp*PETase_M1	
		*Pp*PETase_M1/Phe244Gly	*Pp*PETase_M2	
		*Pp*PETase_M2/Tyr250Gly	*Pp*PETase_M3	
*Ps*PETase	*Piscinibacter sakaiensis*	-	*Ps*PETase_WT	[71]
		Asp186Ala	PETase^A^	
		PETase^A^/Asn233Cys/Ser282Cys	PETase^ACC^	
		PETase^ACC^/Ala179Cys/Ser136Glu/Ser214Thr	PETase^ACCET^	
		PETase^ACCET^/Lys95Asn	PETase^ACCETN^ (Combi-PETase)	
*Sc*PETase	*Streptomyces calvus* DSM 41452	-	*Sc*PETase_WT	[70]
		Ala212Cys/Thr249Cys	*Sc*PETase_M2	
		*Sc*PETase_M1/Asn195His	*Sc*PETase_M3	
		*Sc*PETase_M2/Asn243Lys	*Sc*PETase_M4	
TfCut2	*Thermobifida fusca* KW3	-	TfCut2_WT	[72]
		Ca^2+^/Mg^2+^	TfCut2_cations	
		His184Ser/Phe209Ile	TfCut2_M2a	[73]
		TfCut2_M2a/Gln92Gly	TfCut2_M3a	
		TfCut2_M3a/Ile213Lys	TfCut2_4M_z_	
		Leu32Glu/Ser113Glu	TfCut2_M2b	[74]
		TfCut2_M2b/Thr237Gln	TfCut2_M3b	
V3 PETase	Engineered	Gly119Gln/Asn233Cys/Ser238Trp/Ser282Cys	V3 PETase_WT	[75]
		Lys95Ala	V3 PETase_M1	
		V3 PETase_M1/Arg132Asn	V3 PETase_M2	
		V3 PETase_M2/Arg280Ala	V3 PETase_M3	

**Table 2:** Mutants of PET-degrading enzymes developed through site-directed mutagenesis accompanied by experimental conditions and results.

Enzyme	Mutants	${\Delta T}_{m}$	$T_{m}$	Substrate	Conditions	Degradation Result	Ref.
BhrPETase	His218Ser/Phe222Ile/Ala209Arg/ Asp238Lys/Ala251Cys/ Ala281Cys/Trp104Leu/ Phe243Thr (TurboPETase)	−12 °C	84 °C	Pre-treated PET flakes (11.1% cryst.)	65 °C, pH 8.0, 8 h	98.2% depolymerization	[32]
Est1	Asn213Met/Thr215Pro/Ser115Pro/ Gln93Ala/Leu91Trp (Est_5M)	__	__	Used PET plastic waste (9.1% cryst.)	65 °C, pH 8.0, 72 h	65-fold improvement, 90% degradation	[57]
Est30	Ile171Lys/Met127Ser/Gly130Leu	−10.89 °C	63.18 °C	MHET	50 °C, pH 7.5, 10 min	96.3-fold increase in catalytic efficiency	[58]
LCC	Tyr127Gly/Asp238Cys/Phe243Ile/ Ser283Cys (LCC_ICCG)	+9.3 °C	94.0 °C	Used PET plastic waste	72 °C, pH 8.0, 10 h	>90% depolymerization	[59]
LCC_ICCG	Ser32Leu/Asp18Thr/Ser98Arg/ Thr157Pro/Glu173Gln/Asn213Pro (LCCICCG_I6M)	+1.04 °C	96.13 °C	PET bottle powder (31.30% cryst.)	80 °C, pH 8.0, 24 h	264% increase in soluble products (3.64-fold increase)	[60]
	His218Tyr/Asn248Asp (LCC-A2) His218Tyr/Asn248Asp/Ser247Ala (LCC-A3)	+1.11 °C +0.61 °C	95.25 °C 94.75 °C	Amorphous PET powder (cryst. 8.17%)	72 °C, pH 8.0, 6 h	40.54% increase in product release 39.46% increase in product release	[61]
	His183Tyr/Leu124Gly/Ser29Ala (LCC-YGA)	__	__	Amorphous PET films (cryst. 8%)	70 °C, pH 8.0, 5 h	107% enhancement in hydrolytic activity (2.1-fold improvement)	[62]
PET6	Val91Thr/Ser92Ala (PET6-VSTA)	−0.5 to −1 °C	(56.7–57.2) °C	Used PET plastic waste (cryst. 10%)	50 °C, pH 8.5, 1M NaCl	63% increase in product release	[63]
*Is*PETase	Trp159His/Phe229Tyr	+10.4 °C	61.2 °C	Amorphous PET	40 °C, pH 9.5, 24 h	40-fold increase in total product concentration	[64]
	Ser238Ala Tyr87Glu	+0 °C +12 °C	42 °C 54 °C	PET film	30 °C, pH 7.2, 72 h	~55% increase in product release No product release	[65]
	Ser92Lys/Arg251Ala Ile139Arg	+5.42 °C +8.71 °C	53.08 °C 56.37 °C	PET film (22.3% crystallinity)	40 °C, pH 9.0, 24 h	2.9-fold increase in degradation activity 3.6-fold increase in degradation activity	[66]
	Ile208Val Ser238Tyr Asn212Ala	__	__	PET film (30.88% cryst.)	30 °C, pH 9.4, 72 h	No effect on degradation activity 3.3-fold increase in degradation activity 1.4-fold increase in degradation activity.	[67]
	Ile168Arg/Ser188Asp Ile168Arg/Ser188Glu	+7.4 °C +8.7 °C	50.2 °C 51.5 °C	Amorphous PET film (8.1% cryst.)	40 °C, pH 9.0, 6 d	3.8-fold increase in product concentration 4.3-fold increase in product concentration	[68]
	Asp186Val Asp186Ala Asp186Asn Asp186His	+12.91 °C +11.84 °C +8.89 °C +8.76 °C	59.49 °C 58.42 °C 55.47 °C 55.34 °C	PET film (28.34% cryst.)	40 °C, pH 9.0, 6 d	2.49-fold increase in degradation activity 3.62-fold increase in degradation activity 3.69-fold increase in degradation activity 3.43-fold increase in degradation activity	[69]
*Pp*PETase	Tyr239Arg/Phe244Gly/Tyr250Gly	__	__	PET powder	30 °C, pH 7.0, 24 h	3.1-fold increase in product release	[70]
*Ps*PETase	Asp186Ala/Asn233Cys/Ser282Cys/ Ala179Cys/Ser136Glu/ Ser214Thr/Lys95Asn (Combi-PETase)	+27.2 °C	70.4 °C	PET particles bottle (45% cryst.)	50 °C, pH 9.0, 28 h	4.25-fold increase in degradation activity	[71]
*Sc*PETase	Ala212Cys/Thr249Cys/Asn195His/ Asn243Lys	__	__	PET powder	30 °C, pH 7.0, 24 h	1.9-fold increase in product release	[70]
TfCut2	His184Ser/Gln92Gly/Phe209Ile/ Ile213Lys (4M_z_)	__	__	Amorphous PET film	60 °C, pH 8.0, 96 h	90% degradation rate, 30-fold improvement in catalytic efficiency	[73]
	Leu32Glu/Ser113Glu/Thr237Gln	−0.6 °C	72.2 °C	PET powder (>40% cryst.)	65 °C, pH 8.5, 48 h	5.3-fold depolymerization improvement	[74]
V3 PETase	Lys95Ala/Arg132Asn/Arg280Ala	+3.5 °C	61.25 °C	Pre-treated PET bottle (9% cryst.)	40 °C, pH 8.0, 72 h	3-fold improvement in product release, 100% degradation	[75]

**Table 3:** Molecular dynamics simulation results reported in the reviewed studies. For detailed information regarding the MD parameters see Table S4 in supporting information.

Enzyme	Mutants	RMSF	Hydrogen Bonds	Catalytic Distance	Binding Affinity	Impact ^a^	Ref.
BhrPETase	His218Ser/Phe222Ile/ Ala209Arg/Asp238Lys/ Ala251Cys/Ala281Cys/ Trp104Leu/Phe243Thr (TurboPETase)	Increased at β7-α5 and β8-α6 loop	__	Ser-PET decreased from 4.88 Å to 4.15 Å	__	Activity	[32]
Est1	Asn213Met/Thr215Pro/ Ser115Pro/Gln93Ala/ Leu91Trp (Est_5M)	Decreased at β4-α3, α3-b5, and β8-α6 loops	__	__	__	Activity	[57]
Est30	Ile171Lys/Met127Ser/ Gly130Leu	__	__	Ser-PET < 3.0 Å increased from 11.66% to 34.45%	__	Activity	[58]
LCC	Tyr127Gly/Asp238Cys/ Phe243Ile/Ser283Cys (LCC_ICCG)	__	Increased from 15.2% to 90% of the simulation time between catalytic residues	Ser-His decreased from ~4 Å to 2.8 Å	__	Activity	[59]
LCCICCG	Ser32Leu/Asp18Thr/ Ser98Arg/Thr157Pro/ Glu173Gln/Asn213Pro (LCCICCG_I6M)	Decreased at β8-α6 loop Increased at β7-α5 loop	__	__	__	Stability Activity	[60]
	His218Tyr/Asn248Asp (LCC-A2) His218Tyr/Asn248Asp/ Ser247Ala (LCC-A3)	__	Number of bonds between protein–PET increased from 2.33 to 3.76 (LCC-A2) and 4.79 (LCC-A3)	__	__	Activity	[61]
	His183Tyr/Leu124Gly/ Ser29Ala (LCC-YGA)	Increased at β1-β2 loop and β5 strand	__	__	__	Activity	[62]
PET6	Val91Thr/Ser92Ala (PET6-VSTA)	__	__	His-PET contact frequency increased from 18% to 64%	__	Activity	[63]
*Is*PETase	Trp159His/Phe229Tyr	Decreased between β7-α5 loop and β8 strand	Increased the number of bonds within the enzyme	__	__	Stability	[64]
	Ser238Ala Tyr87Glu	__	__	Ser-His and His-Asp < 3.5 Å increased from 10.1% to 12.8% (Tyr87Glu) and 75.4% (Ser238Ala)	__	Activity	[65]
	Ser92Lys/Arg251Ala Ile139Arg	__	__	Ser-PET decreased from 5.1 Å to 3.4 Å (Ser92Lys/Arg251Ala) and 4.0 Å (Ile139Arg)	__	Activity	[66]
	Ile208Val, Ser238Tyr, Asn212Ala	__	__	__	−25.50 kcal/mol −25.50 kcal/mol −28.36 kcal/mol (−21.20 kcal/mol for wild-type)	Activity	[67]
	Ile168Arg/Ser188Asp Ile168Arg/Ser188Glu	Decreased at α4 helix and β6-β7 loop	__	__	__	Stability	[68]
	Asp186Val, Asp186Ala, Asp186Asn, Asp186His	Decreased at β6-β7 and β7-α5 loops and a5 helix	Occupancy rates increased between Asn/His186-Ser187/188 and between β6-β7 loop and α5/α6 helices	__	−65.27kJ/mol −84.83kJ/mol −93.23 kJ/mol −90.98 kJ/mol (−88.23 kJ/mol for wild-type)	Stability	[69]
*Pp*PETase	Tyr239Arg/Phe244Gly/ Tyr250Gly	Increased at β7-α5, β8-α6 loops, and α6 helix	__	__	__	Activity	[70]
*Ps*PETase	Asp186Ala/Asn233Cys/ Ser282Cys/Ala179Cys/ Ser136Glu/Ser214Thr/ Lys95Asn (Combi-PETase)	__	Break of bond between Ser214Thr and Pro184	__	__	Activity	[71]
*Sc*PETase	Ala212Cys/Thr249Cys/ Asn195His/ Asn243Lys	Increased at the mutation’s locations	__	__	__	Stability	[70]
TfCut2	His184Ser/Gln92Gly/ Phe209Ile/Ile213Lys (4M_z_)	__	__	Ser-PET decreased from 4.6 Å to 3.8 Å	__	Activity	[73]
	Leu32Glu/Ser113Glu/ Thr237Gln	__	__	Ser-PET decreased from 8.2 Å to 3.7 Å	−81.80 kJ/mol (−64.31 kJ/mol for wild-type)	Activity	[74]
V3 PETase	Lys95Ala/Arg132Asn/ Arg280Ala	Decreased at the active site region	__	__	__	Activity	[75]

^a^ The impact refers to the conclusions derived from the MD simulations in terms of if the mutation affected the catalytic activity or thermostability.

**Table 4:** Impact of mutations in the activity and stability of the respective enzymes.

Enzyme	Starting Variant	Added Mutation ^a^	Activity	Stability	Ref.
BhrPETase	BhrPETase_WT	His218Ser/Phe222Ile	↑ ^b^	↓ ^b^	[32]
	BhrPETase_M2	Asp238Lys	↓	↑	
	BhrPETase_M3	Ala251Cys/Ala281Cys	↓	↑	
	BhrPETase_M5	Ala209Arg	↓	↑	
	BhrPETase_M6	Trp104Leu/Phe243Thr	↑	↓	
Est1	Est1_WT	Asn213Met	↑	-	[57]
	Est1_N213M	Thr215Pro	↑	-	
	Est1_N213M/T215P	Ser115Pro	-	↑	
	Est1_MPP	Leu91Trp	↑	-	
	Est1_MPP/L91W	Gln93Ala	↑	-	
Est30	Est_WT	Ile171Lys	↑	↓	[58]
	Est30_M2	Gly130Leu	↑	↑	
	Est30_M8	Met127Ser	↑	↓	
LCC	LCC_WT	Phe243Ile	↑	↓	[59]
	LCC_Μ1	Asp238Cys/Ser283Cys	↓	↑	
	LCC_ICC	Tyr127Gly	↓	↑	
	LCC_ICCG	Asn213Pro	↑ (75 °C)	↑	[60]
	LCC_ICCG	Thr157Pro	↑ (75 °C)	↑	
	LCC_ICCG	Glu173Gln	↑ (75 °C)	-	
	LCC_ICCG	His218Tyr	↑	-	[61]
	LCC_ICCG_Μ1-H218Y	Asn248Asp	↑	-	
	LCC-A2	Ser247Ala	No change	-	
	LCC_ICCG	His183Tyr	↑	-	[62]
	LCC_ICCG_Μ1-H183Υ	Leu124Gly	↑	-	
PET6	PET6_WT	Val91Thr/Ser92Ala	↑	-	[63]
*Is*PETase	*Is*PETase_WT	Trp159His	↑	↑	[64]
	*Is*PETase_M1a	Phe229Tyr	↑	↑	
	*Is*PETase_WT	Ser238Ala	↑	No change	[65]
	*Is*PETase_WT	Tyr87Glu	↓	↑	
	*Is*PETase_WT	Ile139Arg	↑	↑	[66]
	*Is*PETase_M1b	Asp157Glu	↓	↓	
	*Is*PETase_WT	Ser92Lys	↑	↑	
	*Is*PETase_M1c	Arg251Ala	↑	↑	
	*Is*PETase_M1c	Asp157Glu	↓	↓	
	*Is*PETase_WT	Ile208Val	↓	-	[67]
	*Is*PETase_WT	Ser238Tyr	↑	-	
	*Is*PETase_WT	Asn212Ala	↑	-	
	*Is*PETase_WT	Ile168Arg/Ser188Asp	↑	↑	[68]
	*Is*PETase_WT	Ile168Arg/Ser188Glu	↑	↑	
	*Is*PETase_WT	Asp186Val	↑	↑	[69]
	*Is*PETase_WT	Asp186Ala	↑	↑	
	*Is*PETase_WT	Asp186Asn	↑	↑	
	*Is*PETase_WT	Asp186His	↑	↑	
*Pp*PETase	*Pp*PETase_WT	Tyr239Arg	↑	-	[70]
	*Pp*PETase_M1	Phe244Gly/Tyr250Gly	↑	-	
*Ps*PETase	*Ps*PETase_WT	Asp186Ala	↓	↑	[71]
	PETase^A^	Asn233Cys/Ser282Cys	↑	↑	
	PETase^ACC^	Ala179Cys/Ser136Glu/Ser214Thr	↑	↑	
	PETase^ACCET^	Lys95Asn	↑	↑	
*Sc*PETase	*Sc*PETase_WT	Ala212Cys/Thr249Cys	↑	-	[70]
	*Sc*PETase_M2	Asn195His/Asn243Lys	↑	-	
TfCut2	TfCut2_WT	Ca^2+^/Mg^2+^	↑	↑	[72]
	TfCut2_WT	His184Ser/Phe209Ile	↑	-	[73]
	TfCut2_M2a	Gln92Gly /Ile213Lys	↑	-	
	TfCut2_WT	Leu32Glu/Ser113Glu	↑	↑	[74]
	TfCut2_M2b	Thr237Gln	↑	↑	
V3 PETase	V3 PETase_WT	Lys95Ala	↓ (film) ↑ (powder)	↑	[75]
	V3 PETase_M1	Arg132Asn	↑	↓	
	V3 PETase_M2	Arg280Ala	No change	↑	

^a^ Mutation added to the starting variant. ^b^ ↑ indicates an increase and ↓ indicates a decrease.

**Table 5:** Experimental results of the effect of modulation of ionic concentration on enzyme performance.

Enzyme	Ionic Concentration	$T_{m}$	Substrate	Conditions	Degradation Properties	Ref.
*Is*PETase	50 mM NaCl 1 M NaCl	46.2 °C 52.7 °C	Used PET plastic waste (cryst. 10%)	30–50 °C, pH 8.5	21–45 μM at 30–50 °C Decreased at 50 °C 5.6 μM at 30 °C 0.9 μM at 50 °C	[63]
PET6	50 mM NaCl 1 M NaCl	49.8 °C 57.7 °C	Used PET plastic waste (cryst. 10%)	30–50 °C, pH 8.5	0.02–0.2 μM at 30–50 °C 0.08 μM at 40 °C 1.1 μΜ at 50 °C	[63]
TfCut2	None 10 mM MgCl_2_ 10 mM CaCl_2_	71.2 °C 82.0 °C 83.8 °C	PET film	65 °C, pH 8.5	none ~7% weight loss 12.6% weight loss	[72]

## Supplementary Materials

The following supporting information can be downloaded at https://www.mdpi.com/article/10.3390/ijms26167682/s1. Reference [102] is cited in the supplementary materials.
