# Supplementary material for: Atomistic-Level Insights into the Role of Mutations in the Engineering of PET Hydrolases: A Systematic Review
Source: Int J Mol Sci. 2025 Aug 8;26(16):7682. doi: 10.3390/ijms26167682 (PMC12387008; doi:10.3390/ijms26167682)
Supplement: Supplementary file 1 [file ijms-26-07682-s001.zip › ijms-3772110-supplementary.pdf]

Supplementary Material

Atomistic-level Insights into the Role of Mutations in Engineering of PET hydrolases: A Systematic Review

Athina Karaoli,[1,4] Haralampos Tzoupis,[1] Konstantinos D. Papavasileiou,[1,2] Anastasios G. Papadiamantis,[1,2,5] Dimitris G. Mintis,[1,2] Chris T. Kiranoudis,[4] Iseult Lynch,[2,5] Georgia Melagraki,[6] Andreas Afantitis[1,2,3,*]

[1] Department of ChemoInformatics, NovaMechanics Ltd., Nicosia CY-1070, Cyprus; karaoli@novamechanics.com (A.K.); tzoupis@novamechanics.com (H.T.); papavasileiou@novamechanics.com (K.D.P); papadiamantis@novamechanics.com (A.G.P); mintis@novamechanics.com (D.G.M.)
[2] Entelos Institute Ltd, Larnaca 6059, Cyprus
[3] NovaMechanics MIKE, Piraeus 18545, Greece
[4] School of Chemical Engineering, National Technical University, Zografou 15780, Athens, Greece; kyr@chemeng.ntua.gr (C.T.K)
[5] School of Geography, Earth and Environmental Sciences, University of Birmingham, Birmingham B15 2TT, United Kingdom; i.lynch@bham.ac.uk (I.L.)
[6] Division of Physical Sciences and Applications, Hellenic Military Academy, Vari 16672, Greece; georgiamelagraki@gmail.com (G.M.)
*Correspondence: afantitis@novamechanics.com (A.A)

Table S1: PRISMA2020 checklist for systematic reviews

Section and Topic	Item #	Checklist item	Location where item is reported
**TITLE**			
Title	1	Identify the report as a systematic review.	Title (page 1)
**ABSTRACT**			
Abstract	2	See the PRISMA 2020 for Abstracts checklist.	Abstract (page 1)
**INTRODUCTION**			
Rationale	3	Describe the rationale for the review in the context of existing knowledge.	Section 1 (Pages 1-2)
Objectives	4	Provide an explicit statement of the objective(s) or question(s) the review addresses.	Section 2.1 (page 2)
**METHODS**			
Eligibility criteria	5	Specify the inclusion and exclusion criteria for the review and how studies were grouped for the syntheses.	Section 2.2 (page 2-3)
Information sources	6	Specify all databases, registers, websites, organisations, reference lists and other sources searched or consulted to identify studies. Specify the date when each source was last searched or consulted.	Section 2.3 (page 3)
Search strategy	7	Present the full search strategies for all databases, registers and websites, including any filters and limits used.	Section 2.3 (page 3) and Suppl. Material (Table S2)
Selection process	8	Specify the methods used to decide whether a study met the inclusion criteria of the review, including how many reviewers screened each record and each report retrieved, whether they worked independently, and if applicable, details of automation tools used in the process.	Section 2.4 (page 3)
Data collection process	9	Specify the methods used to collect data from reports, including how many reviewers collected data from each report, whether they worked independently, any processes for obtaining or confirming data from study investigators, and if applicable, details of automation tools used in the process.	Section 2.4 (page 3)
Data items	10a	List and define all outcomes for which data were sought. Specify whether all results that were compatible with each outcome domain in each study were sought (e.g. for all measures, time points, analyses), and if not, the methods used to decide which results to collect.	N/A
	10b	List and define all other variables for which data were sought (e.g. participant and intervention characteristics, funding sources). Describe any assumptions made about any missing or unclear information.	N/A
Study risk of bias assessment	11	Specify the methods used to assess risk of bias in the included studies, including details of the tool(s) used, how many reviewers assessed each study and whether they worked independently, and if applicable, details of automation tools used in the process.	N/A
Effect measures	12	Specify for each outcome the effect measure(s) (e.g. risk ratio, mean difference) used in the synthesis or presentation of results.	N/A

Section and Topic	Item #	Checklist item	Location where item is reported
Synthesis methods	13a	Describe the processes used to decide which studies were eligible for each synthesis (e.g. tabulating the study intervention characteristics and comparing against the planned groups for each synthesis (item #5)).	N/A
	13b	Describe any methods required to prepare the data for presentation or synthesis, such as handling of missing summary statistics, or data conversions.	N/A
	13c	Describe any methods used to tabulate or visually display results of individual studies and syntheses.	N/A
	13d	Describe any methods used to synthesize results and provide a rationale for the choice(s). If meta-analysis was performed, describe the model(s), method(s) to identify the presence and extent of statistical heterogeneity, and software package(s) used.	N/A
	13e	Describe any methods used to explore possible causes of heterogeneity among study results (e.g. subgroup analysis, meta-regression).	N/A
	13f	Describe any sensitivity analyses conducted to assess robustness of the synthesized results.	N/A
Reporting bias assessment	14	Describe any methods used to assess risk of bias due to missing results in a synthesis (arising from reporting biases).	N/A
Certainty assessment	15	Describe any methods used to assess certainty (or confidence) in the body of evidence for an outcome.	N/A
**RESULTS**			
Study selection	16a	Describe the results of the search and selection process, from the number of records identified in the search to the number of studies included in the review, ideally using a flow diagram.	Section 3.1 (Pages 3-4)
	16b	Cite studies that might appear to meet the inclusion criteria, but which were excluded, and explain why they were excluded.	Section 3.2 (Page 5-6)
Study characteristics	17	Cite each included study and present its characteristics.	Section 3.4 (Pages 9-25) Tables 2-5)
Risk of bias in studies	18	Present assessments of risk of bias for each included study.	N/A
Results of individual studies	19	For all outcomes, present, for each study: (a) summary statistics for each group (where appropriate) and (b) an effect estimate and its precision (e.g. confidence/credible interval), ideally using structured tables or plots.	N/A
Results of syntheses	20a	For each synthesis, briefly summarise the characteristics and risk of bias among contributing studies.	N/A
	20b	Present results of all statistical syntheses conducted. If meta-analysis was done, present for each the summary estimate and its precision (e.g. confidence/credible interval) and measures of statistical heterogeneity. If comparing groups, describe the direction of the effect.	N/A
	20c	Present results of all investigations of possible causes of heterogeneity among study results.	N/A
	20d	Present results of all sensitivity analyses conducted to assess the robustness of the synthesized results.	N/A
Reporting biases	21	Present assessments of risk of bias due to missing results (arising from reporting biases) for each synthesis assessed.	N/A
Certainty of evidence	22	Present assessments of certainty (or confidence) in the body of evidence for each outcome assessed.	N/A

Section and Topic	Item #	Checklist item	Location where item is reported
**DISCUSSION**			
Discussion	23a	Provide a general interpretation of the results in the context of other evidence.	Section 3.4 (Pages 9-25) Tables 2-5)
	23b	Discuss any limitations of the evidence included in the review.	N/A
	23c	Discuss any limitations of the review processes used.	N/A
	23d	Discuss implications of the results for practice, policy, and future research.	Section 4 (Pages 25-26)
**OTHER INFORMATION**			
Registration and protocol	24a	Provide registration information for the review, including register name and registration number, or state that the review was not registered.	Section 2.1 (Page 2)
	24b	Indicate where the review protocol can be accessed, or state that a protocol was not prepared.	Section 2.1 (Page 2)
	24c	Describe and explain any amendments to information provided at registration or in the protocol.	N/A
Support	25	Describe sources of financial or non-financial support for the review, and the role of the funders or sponsors in the review.	Funding (Page 26)
Competing interests	26	Declare any competing interests of review authors.	Conflicts of Interest (Page 26)
Availability of data, code and other materials	27	Report which of the following are publicly available and where they can be found: template data collection forms; data extracted from included studies; data used for all analyses; analytic code; any other materials used in the review.	N/A

Table S2. Search strategy and query modifications for each database.

Resource	Search Query and Refinements	#studies	Post-query filtering	#studies
Scopus	Search within Article title, Abstract, Keywords:<br>• "PET" OR "polyethylene terephthalate" OR "poly(ethylene terephthalate)"<br>• protein OR enzym*<br>• biodegraded* OR degrad* OR depolymeriz* OR bioconversion<br>• hydrol* OR cataly*<br>• plastic	571	-Type:<br>Articles<br><br>-Language:<br>English	429
PubMed	("PET"[Title/Abstract] OR "polyethylene terephthalate"[Title/Abstract] OR "poly ethylene terephthalate"[Title/Abstract]) AND ("protein"[Title/Abstract] OR "enzym*"[Title/Abstract]) AND ("biodegrad*"[Title/Abstract] OR "degrad*"[Title/Abstract] OR "depolymeriz*"[Title/Abstract] OR "bioconversion"[Title/Abstract]) ("hydrol*"[Title/Abstract] OR "cataly*"[Title/Abstract]) AND plastic[Title/Abstract]	189	-Type:<br>Articles<br><br>-Language:<br>English	140

Table S3: Molecular dynamics simulations results from the reviewed studies along with the conditions under which the simulations were conducted.

Enzyme	Mutants	Conditions	RMSF	Hydrogen bonds	Salt bridges	Catalytic Distance	Binding affinity	Impact[a]	Ref.
BhrPETase	His218Ser/Phe222Ile/ Ala209Arg/Asp238Lys/ Ala251Cys/Ala281Cys/ Trp104Leu/Phe243Thr (TurboPETase)	65°C	Increased at β7-α5 and β8-α6 loop	—	—	Ser-PET decreased from 4.88Å to 4.15Å	—	Activity	[1]
Est1	Asn213Met/Thr215Pro/ Ser115Pro/Gln93Ala/ Leu91Trp (Est_5M)	65°C	Decreased at β4-α3, α3-b5 and β8-α6 loops	—	—	—	—	Activity	[2]
Est30	Ile171Lys/Met127Ser/ Gly130Leu	70°C	—	—	—	Ser-PET < 3.0Å increased from 11.66% to 34.45%	—	Activity	[3]
LCC	Tyr127Gly/Asp238Cys/ Phe243Ile/Ser283Cys (LCCICCG)	60°C	—	Increased from 15.2% to 90% of the simulation time between catalytic residues	—	Ser-His decreased from ~4Å to 2.8Å	—	Activity	[4]
LCCICCG	Ser32Leu/Asp18Thr /Ser98Arg/Thr157Pro/ Glu173Gln/Asn213Pro (LCCICCG_I6M)	37°C	Decreased at β8-α6 loop Increased at β7-α5 loop	—	—	—	—	Stability Activity	[5]
	His218Tyr/Asn248Asp (LCC-A2) His218Tyr/Asn248Asp/ Ser247Ala (LCC-A3)	72°C	—	Number of bonds between protein-PET increased from 2.33 to 3.76 (LCC-A2) and 4.79 (LCC-A3)	—	—	—	Activity	[6]
	His183Tyr/Leu124Gly/ Ser29Ala (LCC-YGA)	70°C	Increased at β1-β2 loop and β5 strand	—	—	—	—	Activity	[7]
PET6	Val91Thr/Ser92Ala (PET6-VSTA)	50°C	—	—	—	His-PET contact frequency increased from 18% to 64%	—	Activity	[8]
*Is*PETase	Trp159His/Phe229Tyr	127°C	Decreased between β7-α5 loop and β8 strand	Increased the number of bonds within the enzyme	Asp118-Arg123 decreased from 5.9Å to 2.7Å	—	—	Stability	[9]

	Ser238Ala<br>Tyr87Glu	30°C	—	—	—	Ser-His and His-Asp < 3.5Å increased from 10.1% to 12.8% (Tyr87Glu) and 75.4% (Ser238Ala)	—	Activity	[10]
	Ser92Lys/Arg251Ala<br>Ile139Arg	25°C	—	—	—	Ser-PET decreased from 5.1Å to 3.4Å (Ser92Lys/Arg251Ala) and 4.0Å (Ile139Arg)	—	Activity	[11]
	Ile208Val,<br>Ser238Tyr,<br>Asn212Ala	27°C	—	—	—	—	-25.50 kcal/mol<br>-25.50 kcal/mol<br>-28.36 kcal/mol for wild-type)	Activity	[12]
	Ile168Arg/Ser188Asp<br>Ile168Arg/Ser188Glu	100°C	Decreased at α4 and β6-β7 loop	—	Arg168 - Glu188 found 4.5Å.<br><br>Arg168 - Asp188 found 5.5Å.	—	—	Stability	[13]
	Asp186Val<br>Asp186Ala<br>Asp186Asn<br>Asp186His	30, 40 and 100°C	Decreased at β6-β7 and β7-α5 loops and a5 helix	Occupancy rates increased between Asn/His186 -Ser187/188 and between β6-β7 loop and α5/α6 helices	—	—	−65.27kJ/mol<br>−84.83kJ/mol<br>−93.23 kJ/mol<br>−90.98 kJ/mol<br>(−88.23 kJ/mol for wild-type)	Stability	[14]
*Pp*PETase	Tyr239Arg/Phe244Gly/<br>Tyr250Gly	27°C	Increased at β7-α5, β8-α6 loops and α6 helix	—	—	—	—	Activity	[15]
*Ps*PETase	Asp186Ala/Asn233Cys/<br>Ser282Cys/Ala179Cys/<br>Ser136Glu/Ser214Thr/<br>Lys95Asn (Combi-PETase)	27°C	—	Break of bond between Ser214Thr and Pro184	—	—	—	Activity	[16]
*Sc*PETase	Ala212Cys/Thr249Cys/<br>Asn195His/ Asn243Lys	27°C	Increased at the mutation’s locations	—	—	—	—	Stability	[15]
TfCut2	His184Ser/Gln92Gly/<br>Phe209Ile/Ile213Lys (4Mz)	60°C	—	—	—	Ser-PET decreased from 4.6Å to 3.8Å	—	Activity	[17]
	Leu32Glu/Ser113Glu/<br>Thr237Gln	65°C	—	—	—	Ser-PET decreased from 8.2Å to 3.7Å	–81.80 kJ/mol<br>(–64.31 kJ/mol for wild-type)	Activity	[18]
V3 PETase	Lys95Ala/Arg132Asn/<br>Arg280Ala	27°C	Decreased at the active site region	—	—	—	—	Activity	[19]

Table S4: Parameters for the MD simulations performed in the various studies reported in this review

Study	Enzyme	Ligand	Enzyme force field	Ligand force field[a]	Temp. (K)	MD length (ns)	Software used	Ref.
Cui et al., 2024	BhRPETase^{WT}<br>TurboPETase	3PET	ff16SB	-	338	100	AMBER 16	[1]
Lu et al., 2024	Est1^{WT}<br>Est_5M	3PET	GROMOS96 54a7	-	338	50	GROMACS 2022.3	[2]
Zhang et al., 2023	Est30^{WT}	MHET	CHARMM36	CGenFF	343	1 and 5	GROMACS 2019.4	[3]
Tournier et al., 2020	LCC^{WT}<br>LCCICCG	protein only<br>2-HE(MHET)$_3$	ff14SB	GAFF	333	100	AMBER 16	[4]
Ding et al., 2023	LCCICCG^{WT}<br>LCCICCG_I6M	protein only	CHARMM	-	310	50	NAMD 2.14b2	[5]
Zheng et al., 2024	LCCICCG^{WT}<br>LCC-A2<br>LCC-A3	3PET	OPLS-AA/M	generated using the LigParGen online tool [6a]	345	20	NAMD 2.12	[6]
Zheng et al., 2024	LCCICCG^{WT}<br>LCC-YGA	2BHET	Amber ff99SB-ildn	-	343	40	GROMACS	[7]
Weigert et al., 2022	PET6^{WT}<br>PET6-VSTA	4PET	CHARMM36	-	323	50	CHARMM	[8]
Meng et al., 2021	*Is*PETase^{WT}<br>*Is*PETase$^{Trp159His/Phe229Tyr}$	protein only	CHARMM22	-	400	20	NAMD 2.12	[9]
Guo et al., 2022	*Is*PETase^{WT}<br>*Is*PETase^{Ser238Ala}<br>*Is*PETase^{Tyr87Glu}	protein only<br>4PET	Amber ff99sb-ildn /<br>OPLS-AA (4PET)	Parametrized via Macromodel (Schrödinger)	300	200 (protein only)<br>5 (with ligand)	GROMACS 2019.3	[10]
Yin et al., 2022	*Is*PETase^{WT}<br>*Is*PETase$^{Ser92Lys/Arg251Ala}$<br>*Is*PETase^{Ile139Arg}	(MHET)$_4$-CH3<br>MHET	-	-	298	50	GROMACS	[11]

Sevilla et al., 2023	*Is*PETase^{WT}<br>*Is*PETase^{Ile208Val}<br>*Is*PETase^{Ser238Tyr}<br>*Is*PETase^{Asn212Ala}	2PET	ff19SB	GAFF2	300	4	AMBER 2022	[12]
Qu et al., 2023	*Is*PETase<br>*Is*PETase$^{Ile168Arg/Ser188Asp}$<br>*Is*PETase$^{Ile168Arg/Ser188Glu}$	protein only	Amber99SB-ILDN	-	373	20	GROMACS 5.1.4	[13]
Qu et al., 2024	*Is*PETase<br>*Is*PETase^{Asp186Val}<br>*Is*PETase^{Asp186Ala}<br>*Is*PETase^{Asp186Asn}<br>*Is*PETase^{Asp186His}	protein only<br>2PET	AMBER99SB-ILDN	-	303 (protein only)<br>303, 313, 403 (with ligand)	50	GROMACS 5.1.4	[14]
Han et al., 2024	*Pp*PETase<br>*Pp*PETase$^{Tyr239Arg/Phe244Gly/Tyr250Gly}$<br>ScPETase<br>ScPETase$^{Ala212Cys/Thr249Cys/Asn195His/Asn243Lys}$	4PET	-	-	300	100	GROMACS 2018.33	[15]
Joho et al., 2024	*Ps*PETase<br>Combi-PETase	8PET	ff14SB	GAFF2	300	1000 (*Ps*PETase)<br>2000 (for Combi-PETase)	AMBER 20	[16]
Chen et al., 2022	TfCut2<br>4Mz	2-HE(MHET)$_5$	Amber ff14SB	GAFF	333	100	AMBER 18	[17]
Meng et al., 2023	TfCut2<br>TfCut2$^{Leu32Glu/Ser113Glu/Thr237Gln}$	2PET	AMBER14	GAFF2	338	50	YASARA	[18]
Then et al., 2015	TfCut2<br>TfCut2 (with Ca^{2+} and Mg^{2+} cations)	protein only	AMBER99SB	-	298	50	GROMACS 4.6	[20]
Ding et al., 2024	*Is*PETase<br>V3 PETase<br>V3 PETase$^{Lys95Ala/Arg132Asn/Arg280Ala}$	protein only<br>PET	ff14SB	GAFF	300	100 (clas. MD)<br>250 (aMD)	AMBER 18	[19]

[a]Values are provided only where the information is available, based on the Methods section in each reference.

References

1. Cui, Y.; Chen, Y.; Sun, J.; Zhu, T.; Pang, H.; Li, C.; Geng, W.-C.; Wu, B. Computational redesign of a hydrolase for nearly complete PET depolymerization at industrially relevant high-solids loading. *Nature Communications* **2024**, *15*, 1417, doi:10.1038/s41467-024-45662-9.
2. Lu, D.; Chen, Y.; Jin, S.; Wu, Q.; Wu, J.; Liu, J.; Wang, F.; Deng, L.; Nie, K. The evolution of cutinase Est1 based on the clustering strategy and its application for commercial PET bottles degradation. *J Environ Manage* **2024**, *368*, 122217, doi:10.1016/j.jenvman.2024.122217.
3. Zhang, J.; Wang, H.; Luo, Z.; Yang, Z.; Zhang, Z.; Wang, P.; Li, M.; Zhang, Y.; Feng, Y.; Lu, D.; et al. Computational design of highly efficient thermostable MHET hydrolases and dual enzyme system for PET recycling. *Commun Biol* **2023**, *6*, 1135, doi:10.1038/s42003-023-05523-5.
4. Tournier, V.; Topham, C.M.; Gilles, A.; David, B.; Folgoas, C.; Moya-Leclair, E.; Kamionka, E.; Desrousseaux, M.L.; Texier, H.; Gavalda, S.; et al. An engineered PET depolymerase to break down and recycle plastic bottles. *Nature* **2020**, *580*, 216-219, doi:10.1038/s41586-020-2149-4.
5. Ding, Z.; Xu, G.; Miao, R.; Wu, N.; Zhang, W.; Yao, B.; Guan, F.; Huang, H.; Tian, J. Rational redesign of thermophilic PET hydrolase LCCICCG to enhance hydrolysis of high crystallinity polyethylene terephthalates. *J Hazard Mater* **2023**, *453*, 131386, doi:10.1016/j.jhazmat.2023.131386.
6. Zheng, Y.; Li, Q.; Liu, P.; Yuan, Y.; Dian, L.; Wang, Q.; Liang, Q.; Su, T.; Qi, Q. Dynamic docking-assisted engineering of hydrolases for efficient PET depolymerization. *ACS Catalysis* **2024**, *14*, 3627-3639, doi:10.1021/acscatal.4c00400. a. Doddam LS.; Cabeza de Vaca, I.; Tirado-Rives J.; Jorgensen, WL.; LigParGen web server: an automatic OPLS-AA parameter generator for organic ligands. *Nucleic Acids Research* **2017,** *45(Issue W1),* W331-W336, doi:10.1093/nar/gkx312
7. Zheng, Y.; Zhang, J.; You, S.; Lin, W.; Su, R.; Qi, W. Efficient thermophilic PET hydrolase enhanced by cross correlation-based accumulated mutagenesis strategy. *Bioresource Technology* **2024**, 130929, doi:10.1016/j.biortech.2024.130929.
8. Weigert, S.; Perez-Garcia, P.; Gisdon, F.J.; Gagsteiger, A.; Schweinshaut, K.; Ullmann, G.M.; Chow, J.; Streit, W.R.; Hocker, B. Investigation of the halophilic PET hydrolase PET6 from Vibrio gazogenes. *Protein Sci* **2022**, *31*, e4500, doi:10.1002/pro.4500.
9. Meng, X.; Yang, L.; Liu, H.; Li, Q.; Xu, G.; Zhang, Y.; Guan, F.; Zhang, Y.; Zhang, W.; Wu, N.; et al. Protein engineering of stable IsPETase for PET plastic degradation by Premuse. *Int J Biol Macromol* **2021**, *180*, 667-676, doi:10.1016/j.ijbiomac.2021.03.058.
10. Guo, B.; Vanga, S.R.; Lopez-Lorenzo, X.; Saenz-Mendez, P.; Ericsson, S.R.; Fang, Y.; Ye, X.; Schriever, K.; Backstrom, E.; Biundo, A. Conformational selection in biocatalytic plastic degradation by PETase. *Acs Catalysis* **2022**, *12*, 3397-3409, doi:10.1021/acscatal.1c05548.
11. Yin, Q.; You, S.; Zhang, J.; Qi, W.; Su, R. Enhancement of the polyethylene terephthalate and mono-(2-hydroxyethyl) terephthalate degradation activity of Ideonella sakaiensis PETase by an electrostatic interaction-based strategy. *Bioresour Technol* **2022**, *364*, 128026, doi:10.1016/j.biortech.2022.128026.
12. Sevilla, M.E.; Garcia, M.D.; Perez-Castillo, Y.; Armijos-Jaramillo, V.; Casado, S.; Vizuete, K.; Debut, A.; Cerda-Mejia, L. Degradation of PET Bottles by an Engineered Ideonella sakaiensis PETase. *Polymers (Basel)* **2023**, *15*, 1779, doi:10.3390/polym15071779.
13. Qu, Z.; Chen, K.; Zhang, L.; Sun, Y. Computation-Based Design of Salt Bridges in PETase for Enhanced Thermostability and Performance for PET Degradation. *Chembiochem* **2023**, *24*, e202300373, doi:10.1002/cbic.202300373.
14. Qu, Z.; Zhang, L.; Sun, Y. Molecular Insights into the Enhanced Activity and/or Thermostability of PET Hydrolase by D186 Mutations. *Molecules* **2024**, *29*, 1338, doi:10.3390/molecules29061338.
15. Han, Z.; Nina, M.R.H.; Zhang, X.; Huang, H.; Fan, D.; Bai, Y. Discovery and characterization of two novel polyethylene terephthalate hydrolases: One from a bacterium identified in human feces and one from the Streptomyces genus. *J Hazard Mater* **2024**, *472*, 134532, doi:10.1016/j.jhazmat.2024.134532.
16. Joho, Y.; Royan, S.; Caputo, A.T.; Newton, S.; Peat, T.S.; Newman, J.; Jackson, C.; Ardevol, A. Enhancing PET Degrading Enzymes: A Combinatory Approach. *Chembiochem* **2024**, *25*, e202400084, doi:10.1002/cbic.202400084.
17. Chen, X.Q.; Guo, Z.Y.; Wang, L.; Yan, Z.F.; Jin, C.X.; Huang, Q.S.; Kong, D.M.; Rao, D.M.; Wu, J. Directional-path modification strategy enhances PET hydrolase catalysis of plastic degradation. *J Hazard Mater* **2022**, *433*, 128816, doi:10.1016/j.jhazmat.2022.128816.

18. Meng, S.; Li, Z.; Zhang, P.; Contreras, F.; Ji, Y.; Schwaneberg, U. Deep learning guided enzyme engineering of Thermobifida fusca cutinase for increased PET depolymerization. *Chinese Journal of Catalysis* **2023**, *50*, 229-238, doi:10.1016/S1872-2067(23)64470-5.
19. Ding, K.; Levitskaya, Z.; Sana, B.; Pasula, R.R.; Kannan, S.; Adam, A.; Sundaravadanam, V.V.; Verma, C.; Lim, S.; Ghadessy, J.F. Modulation of PETase active site flexibility and activity on morphologically distinct polyethylene terephthalate substrates by surface charge engineering. *Biochemical Engineering Journal* **2024**, *209*, 109420, doi:10.1016/j.bej.2024.109420.
20. Then, J.; Wei, R.; Oeser, T.; Barth, M.; Belisario-Ferrari, M.R.; Schmidt, J.; Zimmermann, W. Ca2+ and Mg2+ binding site engineering increases the degradation of polyethylene terephthalate films by polyester hydrolases from Thermobifida fusca. *Biotechnol J* **2015**, *10*, 592-598, doi:10.1002/biot.201400620
